# Urban-rural differences in the association between long-term exposure to ambient particulate matter (PM) and malnutrition status among children under five years old: A cross-sectional study in China

**DOI:** 10.7189/jogh.13.04112

**Published:** 2023-09-22

**Authors:** Xianzhi Li, Yajie Li, Bin Yu, Qucuo Nima, Haorong Meng, Meiying Shen, Zonglei Zhou, Shunjin Liu, Yunyun Tian, Xiangyi Xing, Li Yin

**Affiliations:** 1 Meteorological Medical Research Center, Panzhihua Central Hospital, Panzhihua, Sichuan Province, China; 2 Clinical Medical Research Center, Panzhihua Central Hospital, Panzhihua, Sichuan Province, China; 3 Dali University, Dali, Yunnan Province, China; 4 Tibet Center for Disease Control and Prevention, Lhasa, Tibet Autonomous Region, China; 5 Institute for Disaster Management and Reconstruction, Sichuan University - Hong Kong Polytechnic University, Chengdu, Sichuan Province, China; 6 Yunnan Center for Disease Control and Prevention, Kunming, Yunnan Province, China; 7 Nursing department, Panzhihua Central Hospital, Panzhihua, Sichuan Province, China; 8 Department of Epidemiology, School of Public Health, Fudan University, Shanghai, China; 9 Department of Pharmacy, Panzhihua Central Hospital, Panzhihua, Sichuan Province, China

## Abstract

**Background:**

The evidence regarding the relationship between postnatal exposure of air pollution and child malnutrition indicators, as well as the corresponding urban-rural disparities, is limited, especially in low-pollution area of low- and middle-income countries (LMICs). Therefore, our aim was to contrast the effect estimates of varying ambient particulate matter (PM) on malnutrition indicators between urban and rural areas in Tibet, China.

**Methods:**

Six malnutrition indicators were evaluated in this study, namely, Z-scores of height for age (HFA), Z-scores of weight for age (WFA), Z-scores of weight for height (WFH), stunting, underweight, and wasting. Exposure to particles with an aerodynamic diameter ≤2.5 micron (μm) (PM_2.5_), particles with an aerodynamic diameter ≤10 μm (PM_10_) and particles with an aerodynamic diameter between 2.5 and 10 μm (PM_c_) was estimated using satellite-based random forest models. Linear regression and logistic regression models were used to assess the associations between PM and the above malnutrition indicators. Furthermore, the effect estimates of different PM were contrasted between urban and rural areas.

**Results:**

A total of 2511 children under five years old were included in this study. We found long-term exposure to PM_2.5_, PM_c_, and PM_10_ was associated with an increased risk of stunting and a decreased risk of underweight. Of these air pollutants, PM_c_ had the strongest association for Z-scores of HFA and stunting, while PM_2.5_ had the strongest association for underweight. The results showed that the odds ratio (OR) for stunting were 1.36 (95% confidence interval (CI) = 1.06 to 1.75) per interquartile range (IQR) microgrammes per cubic metre (μg/m^3^) increase in PM_2.5_, 1.80 (95% CI = 1.30 to 2.50) per IQR μg/m^3^ increase in PM_c_ and 1.55 (95% CI = 1.17 to 2.05) per IQR μg/m^3^ increase in PM_10_. The concentrations of PM were higher in urban areas, and the effects of PM on malnutrition indicators among urban children were higher than those of rural children.

**Conclusions:**

Our results suggested that PM exposure might be an important trigger of child malnutrition. Further prospective researches are needed to provide important scientific literature for understanding child malnutrition risk concerning postnatal exposure of air pollutants and formulating synthetically social and environmental policies for malnutrition prevention.

Child malnutrition remains a major public health crisis globally [1]. According to World Health Organization (WHO) reports, more than 149.2 million children under five years old suffer from malnutrition and the majority of these come from low- and middle-income countries (LMICs) [2,3]. Though the prevalence of malnutrition under five years old has decreased in China in the past decade, it remains high in poor rural counties [3,4]. It is well established that early-life malnutrition was associated with lower productivity and earnings in adulthood, increased risk of morbidity and mortality, and adverse cognitive health later in life [2,5-7].

Poor water, hygiene conditions and sanitation are recognised as a major cause of child malnutrition [8]. However, large high quality studies found the prevalence of malnutrition failed to be improved through water, hygiene conditions and sanitation interventions [9-11]. These results highlight a broader view of environmental factors that might affect child malnutrition is needed [12]. Ambient air pollution, as an important and widespread environmental exposure factor (about 98% children under five years old are exposed to exceeding air pollution concentrations [13]), may affect child growth by impairing immune development and function, inducing clinical and subclinical infection, altering dietary intake and metabolism, leading to vitamin D deficiency, etc. [14]. Yet, compared with water, hygiene conditions and sanitation, the potential effect of air pollution on child malnutrition has received little attention [14]. Thus, increased attention is urgently needed to define the effect of air pollution on child malnutrition during the early years of life. An improved understanding of these relationships is necessary for the development of new intervention strategies, which would contribute to a comprehensive approach that addresses multiple causal factors for the prevention of child malnutrition such as stunting [14].

Most of the previous evidence for a link between air pollution and child growth focused mainly on prenatal exposure to air pollution and adverse birth outcomes such as early foetal loss, small for gestational age, preterm birth and low birthweight [15-17]. A few studies used postnatal household air pollution as main exposure of interest and found postnatal exposure to household air pollution was inversely associated with Z-scores of height-for-age (HFA) and stunting [18].

Very few studies have explored the effect of postnatal exposure of ambient air pollution on postnatal growth, such as stunting [1]. A study conducted in Bangladesh (annual ambient fine particulate matter particles with an aerodynamic diameter ≤2.5 micron (μm) (PM_2.5_)) level >46 microgrammes per cubic metre (μg/m^3^)) found significant increases in the relative risk of child stunting, wasting, and underweight with higher levels of exposure to PM_2.5_ [19]. Another study included 218 152 children under five from India (average concentration of PM_2.5_ = 55 μg/m^3^) found a 100 μg/m^3^ increase in ambient PM_2.5_ in early-life was associated with a 0.05 (95% confidence interval (CI) = 0.01 to 0.09) standard deviation (SD) reduction in child height [20]. A final study conducted in 32 countries in Africa (average concentration of PM_2.5_ = 35.7 μg/m^3^) found early-life ambient PM_2.5_ exposure was associated with Z-scores of HFA (beta (β) = -0.033, 95% CI = -0.059 to -0.008), and indications of a general trend of a positive association with stunting (odds ratio (OR) = 1.024, 95% CI = 0.991 to 1.059) [1]. Studies mentioned above provided limited information on the association between air pollution PM with different particle sizes and malnutrition. Furthermore, all of these studies were conducted in relatively high-pollution areas. There is little evidence for a threshold for air pollution below which no harmful health effects could be anticipated [21,22]. Previous studies found that harmful health effects of air pollution may be more pronounced in low-pollution area [21,23]. Thus, more studies are needed to explore relationships between postnatal exposure of ambient air pollution and postnatal growth in low-pollution areas. Finally, previous studies have failed to address the association between ambient air pollution and malnutrition in comparable urban and rural areas.

Located on the Tibetan Plateau in southwest China, Tibet is famous for its high altitude and good air quality, which makes it a good site for studying the health effects of air pollution in low-pollution areas. Besides, it has been speculated that the difference in air pollutants component, climate conditions and population adaptability in different regions may lead to the difference in health effects of air pollutants [24,25]. Studies focusing on the effect of air pollution on malnutrition are urgently needed to assess whether this effect exists and its size in Tibet.

To fill these gaps, this study aimed to assess the impacts of ambient air PM (PM_2.5_, particulate matter with aerodynamic diameters between 2.5 to 10 μm (PM_c_) and particulate matter with aerodynamic diameters ≤10 μm (PM_10_)) on six malnutrition indicators (including Z-scores of HFA, Z-scores of weight for age (WFA), Z-scores of weight for height (WFH), stunting, underweight and wasting) among children under five years old in Tibet. In addition, we further contrasted the effect of ambient air PM with different particle sizes. Finally, we explored residence as potential effect modifier in the association between ambient air PM and malnutrition indicators. We hypothesised that PM was associated with unfavourable malnutrition indicators among Tibetan children under five years old, and that this effect is more pronounced in urban children than in rural children.

## METHODS

### Study population

A detailed cross-sectional study and children’s information were previously presented [26]. Specifically, a three-stage, stratified, cluster sampling was employed to select eligible individuals in Tibet Autonomous Region of Southwest China from July to October 2020. In brief, a total of eight counties in Tibet were first selected proportional to population size and then five towns or subdistricts from each county were selected as the primary sampling units. Four villages or communities were randomly selected for each of the forty primary sampling units. Furthermore, a structured questionnaire was used to interview both children aged 0-71 months and their parents living in selected villages or communities. Finally, a total of 3048 children were included in this survey.

The exclusion criteria of this study were as follows: (1) non-Tibetan children; (2) children who had lived at the survey site for less than 12 months; (3) children without available information on any outcome, exposure or adjusted covariables; and (4) children aged under 12 months. We excluded children aged under 12 months to better control the influence of dietary factors and feeding practices on the associations between PM and malnutrition indicators. Ultimately, 2511 children were included in this analysis, with an enrolment rate of 82.4% (Figure S1 in the **Online Supplementary Document**).

### Data collection

We collected baseline information on children’s demographic characteristics (age, sex, ethnic group, residence, low birth weight, annual household income, drinking water source), history of illness (asthma history, anaemia history, history of dental, being ill for the last two weeks), feeding practices (early initiation of breastfeeding (within one hour of birth), exclusive breastfeeding under six months, continued breastfeeding at one year, introduction of complementary foods between six and eight months of age, dietary diversity, meal frequency, consumption of iron-rich or iron-fortified foods), secondary smoking exposure, and maternal demographic characteristics (education level, height, weight, anaemia history during pregnancy) were collected by using a structured questionnaire. The detailed definition and measurement of covariates are presented as follows: (a) age – queried the child's birth registration information and measured in months. (b) Sex – males and females. (c) Asthma history, anaemia history and history of dental caries – assessed whether the child had asthma, anaemia or dental caries prior to the data collection date by guardian self-declaration and categorised into “yes”, “no” and “not sure”. (d) Low birth weight – obtained the child's birth weight by asking the guardian, and defined low birth weight as a birth weight of less than 2500 grammes and categorised into “yes”, “no” and “not sure”. (e) Optimal feeding practice scores – 24 hours dietary recall method was used to collect information on dietary practice by asking the guardian to analyse dietary diversity and meal frequency. According to the WHO Infant and Young Children Feeding Practice Guidelines [27], the minimum dietary diversity was met if a child took four or more of the seven food groups (including (i) grains, roots and tubers, (ii) legumes and nuts, (iii) dairy products, (iv) flesh foods, (v) eggs, (vi) vitamin A-rich fruits and vegetables, (vii) other fruits and vegetables) on the previous day; the minimum meal frequency was met if a child ate meal more than three times on the previous day. Besides, data on breast-feeding and complementary food feeding were also collected, including (i) whether the child was breastfed within one hour of birth, (ii) whether the child was fed exclusively with breast milk until six months of age, (iii) whether the child was given complementary foods between six and eight months of age, (iv) whether the child was consistently breastfed until 12 months of age, and (v) whether the child was fed an Fe-rich food or Fe-fortified food on the previous day. For the above-mentioned seven items, we assigned each item a score of zero or one. Then, we summed the scores for the seven items to obtain an optimal feeding practice score. Optimal feeding practice scores were divided into low and high scores based on the median feeding practice scores (median = 3). (f) Maternal educational level: obtained by asking the child's guardian, and assessed by the highest educational level completed by the mother of the child and categorised into illiterate, primary school and junior high school or above. (g) Maternal height: measured objectively in centimetres and categorised into three groups: <160.0 cm, 160.0-169.9 cm and ≥170.0 cm. The maternal height categories were adapted from several earlier studies [28-30]. (h) Maternal weight: measured objectively in kilogrammes (kg) and categorised into three groups: <50.0 kg, 50.0 ∼ 59.9 kg and ≥60.0 kg. The weight categories were adapted from previous study [31]. (i) Mother suffering from anaemia during pregnancy: assessed by whether the mother suffered from anaemia during pregnancy and categories into yes, no and not sure. (j) Wealth status: assessed by self-report yearly family income and categorised into five groups: poorest (<12 000 Chinese Yuan (CNY)), poorer (12 000-19 999 CNY), middle (20 000-39 999 CNY), richer (40 000-59 999 CNY) and richest (>60 000 CNY). (k) Drinking water source: assessed by the type of water source used by the household and dichotomised into improved and unimproved. According to WHO guideline [32], improved water sources referred to piped water and protected wells, and unimproved water sources referred to springs, lakes, ponds, unprotected wells, rivers and dams. (l) Residence: assessed by the place of residence and dichotomised into rural areas and urban areas based on the urban–rural classification code formulated by the National Bureau of Statistics of the People’s Republic of China (2020). (m) Secondary smoke: defined as whether child had a passive smoking history at least once a week by guardian self-declaration and dichotomised into yes and No. (n) Altitude: measured and recorded the altitude and geographical location of survey spots by Global Positioning System (GPS). (o) Relative humidity, mean temperature: obtained from National Earth System Science Data Center, National Science & Technology Infrastructure of China (http://www.geodata.cn) and matched according to the latitude and longitude of the child's residence.

The anthropometric measurements of malnutrition included children height and weight. Height was measured with children’s shoes off in a recumbent position (for children younger than two years of age) or standing position (for children older than two years of age) three times. Weight was measured three times using a weight measurement device, with children wearing light clothing and bare feet. Height and weight were calculated by averaging the above measurements, respectively.

### Outcome assessment

The Z-scores were calculated by dividing the difference between the observed value and the mean value of the reference population by the SD of the reference population. By calculating Z-scores, stunting (Z-scores<-2 for HFA), underweight (weight-for-age (WFA)) and wasting (weight-for-height (WFH)) were determined according to the WHO’s 2006 Child Growth Standard [33], respectively. Stunting, underweight, and wasting were primary outcome; and Z-scores of HFA, Z-scores of WFA, and Z-scores of WFH were secondary outcome.

### Exposure

PM_2.5_ and PM_10_ data were obtained from the ChinaHighAirPollutionts (CHAP) Data set (https://weijing-rs.github.io/product.html, accessed data: 9 December 2022). Based on monitoring data, satellite remote sensing, temperature, humidity and land use information, and other spatial and temporal predictors, a space-time extremely randomised trees (STET) model was employed to estimated PM_2.5_ and PM_10_ concentrations at a 1 kilometre (km) × 1 km spatial resolution. The model shows a high predictive ability and is robust to noise [34-36]. The 10-fold cross-validation R^2^ (root mean square error) for the daily prediction of PM_2.5_ and PM_10_ were 0.92 (10.76 μg/m^3^) [34,35] and 0.90 (21.12 μg/m^3^) [36], respectively. The one-year average concentration of individual PM_2.5_ and PM_10_ exposure was calculated according to geocoded residential addresses. The one-year average concentration of individual PM_c_ exposure was calculated as the difference between PM_10_ and PM_2.5_.

### Statistical analysis

We used multivariable linear regression models to explore the long-term effects of PM exposure on Z-scores of HFA, Z-scores of WFA, Z-scores of WFH, and the effect estimates were expressed as β and 95% CI. We used multivariable logistic regression models to assess the association between PM exposure and the risk of stunting, underweight, and wasting, and the effect estimates were expressed as OR and 95% CI. We estimated the unadjusted models (Table S1 and S2 in the **Online Supplementary Document**) and the main models that were adjusted for the confounding variables including age, sex, low birth weight, asthma history, anaemia history, history of dental caries, being ill for the last two weeks, optimal feeding scores, secondary smoke exposure, residence, maternal education level, maternal height, maternal weight, mother suffering from anaemia during pregnancy, wealth category, drinking water source, relative humidity, mean temperature and altitude. All associations were reported per 10 μg/m^3^ increase of PM and per IQR μg/m^3^ increase of PM. An increase of 10 μg/m^3^ of PM makes comparisons with other studies possible. And an increase of IQR μg/m^3^ of PM can help us to compare the long-term effect of different particle sizes of PM. Besides, the nonlinear relationship between PM and malnutrition indicators was explored by using restricted cubic spline analysis.

To examine whether the associations were consistent among different subpopulations, subgroup analyses were performed by children residence (rural areas vs. urban areas), sex (male vs. female), age (<36 vs. ≥36 months), wealth status (low income vs. high income), drinking water source (improved vs. unimproved) and optimal feeding scores (high scores vs. low scores). Z test was used to test for statistically significant difference in effect estimates (β or OR) across categories within subgroups; for example, for continuous outcome variable in rural area and urban area, we calculated:



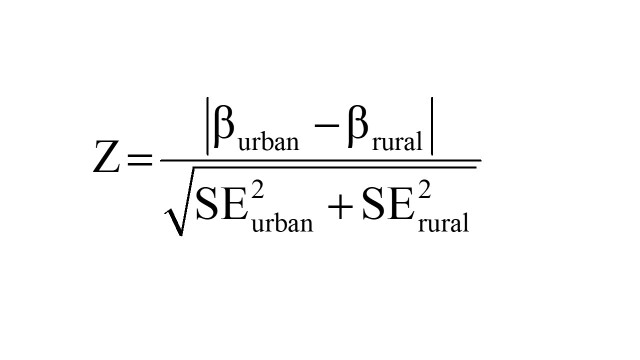



for categorical outcome variable in rural area and urban area, we calculated:



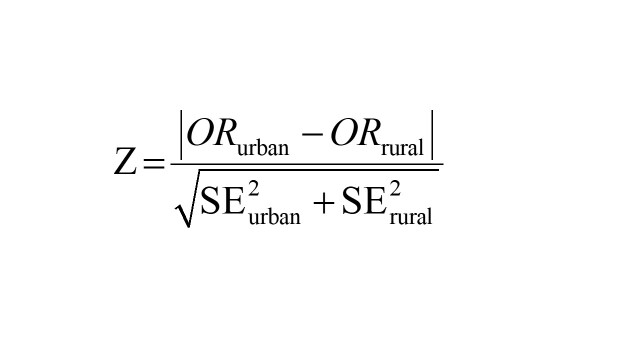



We also performed sensitivity analyses. To test the robustness of our results, three months, six months, nine months and 12 months (one year) average ambient PM concentrations were used to fit the adjusted models.

All statistical analyses were performed using R (version 4.2.2), and statistical significance was declared if *P* < 0.05.

## RESULTS

### Demographic characteristics

**Table 1** described the characteristics of 2511 children aged 0-71 months in this study. Among these children, 2065 and 446 lived in rural area and urban area, respectively. Compared with rural children, urban children were more likely to be older, suffering from asthma, suffering from dental caries, eating Fe-rich or Fe-fortified food, and exposed to secondary smoke, with a lower low birth weight rate, higher dietary diversity, better wealth status and cleaner drinking water source. The mothers of urban children tended to have better education levels, higher height, heavier weight and suffer from anaemia during pregnancy than their counterparts. The children in rural areas were more likely to be breastfed within 1 hour after delivery, breastfed exclusively until 6 months, given complementary foods between six and eight months of age and consistently breastfed until 12 months.

**Table 1:** Basic characteristics of study participants

Variables	Total (n = 2511)	Rural area (n = 2065)	Urban area (n = 446)	*P-*value
**Sex, n (%)**				
Female	1296 (51.6%)	1069 (51.8%)	227 (50.9%)	0.750
Male	1215 (48.4%)	996 (48.2%)	219 (49.1%)	
**Age in months, n (%)**				
<36	964 (38.4%)	947 (45.9%)	17 (3.8%)	<0.001
≥36	1547 (61.6%)	1118 (54.1%)	429 (96.2%)	
**Low birth weight, n (%)**				
Yes	143 (5.7%)	125 (6.0%)	18 (4.0%)	0.006
No	2110 (84.0%)	1713 (83.0%)	397 (89.0%)	
Not sure	258 (10.3%)	227 (11.0%)	31 (7.0%)	
**Asthma history, n (%)**				
Yes	37 (1.5%)	30 (1.5%)	7 (1.6%)	0.036
No	2291 (91.2%)	1872 (90.6%)	419 (93.9%)	
Not sure	183 (7.3%)	163 (7.9%)	20 (4.5%)	
**Anaemia history, n (%)**				
Yes	44 (1.8%)	36 (1.7%)	8 (1.8%)	0.051
No	2282 (90.8%)	1865 (90.4%)	417 (93.5%)	
Not sure	185 (7.4%)	164 (7.9%)	21 (4.7%)	
**Suffering from dental caries, n (%)**				
Yes	136 (5.4%)	99 (4.8%)	37 (8.3%)	0.010
No	2240 (89.2%)	1850 (89.6%)	390 (87.4%)	
Not sure	135 (5.4%)	116 (5.6%)	19 (4.3%)	
**Being ill for the last two weeks, n (%)**				
Yes	259 (10.3%)	207 (10.0%)	52 (11.7%)	0.300
No	2252 (89.7%)	1858 (90.0%)	394 (88.3%)	
**Child breastfed within one hour after delivery, n (%)**				
Yes	184 (7.3%)	182 (8.8%)	2 (0.4%)	<0.001
No	736 (29.3%)	721 (34.9%)	15 (3.4%)	
Not sure	1591 (63.4%)	1162 (56.3%)	429 (96.2%)	
**Exclusive breast feeding until six months, n (%)**				
Yes	969 (38.6%)	825 (40.0%)	144 (32.3%)	0.010
No	1510 (60.1%)	1214 (58.8%)	296 (66.4%)	
Not sure	32 (1.3%)	26 (1.2%)	6 (1.3%)	
**Child given complementary foods between six and eight months of age, n (%)**				
Yes	677 (27.0%)	576 (27.9%)	101 (22.6%)	0.022
No	1772 (70.6%)	1434 (69.4%)	338 (75.8%)	
Not sure	62 (2.4%)	55 (2.7%)	7 (1.6%)	
**Child consistently breastfed until 12 months, n (%)**				
Yes	1403 (55.9%)	1240 (60.0%)	163 (36.5%)	<0.001
No	1108 (44.1%)	825 (40.0%)	283 (63.5%)	
**Dietary diversity, n (%)**				
<4	476 (19.0%)	450 (21.8%)	26 (5.8%)	<0.001
≥4	2035 (81.0%)	1615 (78.2%)	420 (94.2%)	
**Fe-rich or Fe-fortified food, n (%)**				
Yes	150 (6.0%)	96 (4.6%)	54 (12.1%)	<0.001
No	2361 (94.0%)	1969 (95.4%)	392 (87.9%)	
**Meal frequency, times, n (%)**				
<3	62 (2.5%)	45 (2.2%)	17 (3.8%)	0.062
≥3	2449 (97.5%)	2020 (97.8%)	429 (96.2%)	
**Secondary smoke, n (%)**				
Yes	641 (25.5%)	440 (21.3%)	201 (45.1%)	<0.001
No	1870 (74.5%)	1625 (78.7%)	245 (54.9%)	
**Maternal education level, n (%)**				
Illiteracy	1272 (50.7%)	1243 (60.2%)	29 (6.5%)	<0.001
Primary school	429 (17.0%)	403 (19.5%)	26 (5.8%)	
Junior high school and above	810 (32.3%)	419 (20.3%)	391 (87.7%)	
**Maternal height in cm, n (%)**				
<160.0	703 (28.0%)	625 (30.3%)	78 (17.5%)	<0.001
160.0 ~ 169.9	1676 (66.7%)	1339 (64.8%)	337 (75.5%)	
≥170.0	132 (5.3%)	101 (4.9%)	31 (7.0%)	
**Maternal weight, kg, n (%)**				
<50.0	493 (19.6%)	417 (20.2%)	76 (17.0%)	<0.001
50.0 ~ 59.9	1318 (52.5%)	1138 (55.1%)	180 (40.4%)	
≥60.0	700 (27.9%)	510 (24.7%)	190 (42.6%)	
**Mother suffering from anaemia during pregnancy, n (%)**				
Yes	229 (9.1%)	180 (8.7%)	49 (11.0%)	<0.001
No	1919 (76.4%)	1559 (75.5%)	360 (80.7%)	
Not sure	363 (14.5%)	326 (15.8%)	37 (8.3%)	
**Wealth status**				
Poorest	463 (18.4%)	422 (20.4%)	41 (9.2%)	<0.001
Poorer	1030 (41.0%)	956 (46.3%)	74 (16.6%)	
Middle	572 (22.8%)	511 (24.7%)	61 (13.7%)	
Richer	180 (7.2%)	115 (5.6%)	65 (14.6%)	
Richest	266 (10.6%)	61 (3.0%)	205 (46.0%)	
**Drinking water source**				
Improved	1867 (74.4%)	1459 (70.7%)	408 (91.5%)	<0.001
Unimproved	644 (25.6%)	606 (29.3%)	38 (8.5%)	

cm – centimetre, kg – kilogramme

**Table 2** shows the one-year average concentrations of PM_2.5_, PM_c_, PM_10_ at which the children lived and malnutrition indicators. The interquartile range (IQR) of PM_2.5_, PM_c_, PM_10_ were 5.59 μg/m^3^, 5.41 μg/m^3^, and 10.62 μg/m^3^, respectively. The concentrations of ambient PM in urban areas were higher than those in rural areas (*P_urban-rural_* for PM_2.5_<0.001, *P_urban-rural_* for PM*_c_* = 0.003, *P_urban-rural_* for PM_10_ = 0.032). The Z-scores of HFA and WFA among urban children were higher than among rural children (*P* < 0.001). The prevalence rates of stunting in urban areas were lower than in rural areas (*P* < 0.001).

**Table 2:** Descriptive one-year average concentrations of particulate matter (PM) and malnutrition indicators of children by residence

Variables	Total	Urban area	Rural area	*P*-value
	**Median (Q1, Q3)**	**Median (Q1, Q3)**	**Median (Q1, Q3)**	
PM_2.5_ (μg/m^3^)	12.67 (11.17-16.76)	13.00 (11.20-16.90)	12.30 (10.40-16.60)	<0.001
PM_c_ (μg/m^3^)	15.81 (13.52-18.93)	15.90 (12.70-19.00)	15.40 (15.40-18.90)	0.003
PM_10_ (μg/m^3^)	27.48 (24.88-35.50)	28.50 (24.50-36.00)	25.80 (25.80-35.50)	0.032
Z-scores of HFA	-0.33 (-1.42,0.15)	0.15 (-0.36, 0.70)	-0.54 (-1.58, 0.12)	<0.001
Z-scores of WFA	-0.82 (-2.16,0.13)	-0.38 (-2.16, 0.75)	-0.91 (-2.14, 0.02)	<0.001
Z-scores of WFH	0.01 (-0.79,0.82)	-0.01 (-0.66, 0.74)	0.01 (-0.81, 0.83)	0.601
	**N (%)**	**N (%)**	**N (%)**	***P*-value**
Stunting*				<0.001
Yes	376 (15.0)	14 (3.1)	362 (17.5)	
No	2135 (85.0)	432 (96.9)	1703 (82.5)	
**Underweight†**				1.000
Yes	668 (26.6)	118 (26.5)	550 (26.6)	
No	1843 (73.4)	328 (73.5)	1515 (73.4)	
**Wasting‡**				0.660
Yes	155 (6.2)	25 (5.6)	130 (6.3)	
No	2356 (93.8)	421 (94.4)	1935 (93.7)	

PM_2.5_ – particulate matter with an aerodynamic diameter of 2.5 micron (μm), PM_c_ – particulate matter with an aerodynamic diameter of 2.5 to 10 μm, PM_10_ – particulate matter with an aerodynamic diameter of 10 μm, HFA – height for age, WFA – weight for age, WFH – weight for height

*Stunting: Z-scores of HFA<-2.

†Underweight: Z-scores of WFA<-2.

‡Wasting: Z-scores of WFH<-2.

### Associations between ambient air pollutant exposure and malnutrition indicators

**Table 3** showed effect estimates and 95% CI for the association between the malnutrition indicators and a 10 μg/m^3^ increase in average one-year PM_2.5_, PM_c_, and PM_10_ exposure according to the adjusted models. In brief, significant changes statistically in Z-scores of HFA, stunting, and underweight were observed per 10 increments in the PM_2.5_, PM_c_, and PM_10_ concentrations. For example, each 10 μg/m^3^ increase in PM_2.5_ was associated with decreased Z-scores of HFA (β = -0.23, 95% CI = -0.42 to -0.05), OR for stunting of 1.74 (95% CI = 1.11 to 2.72), and OR for underweight of 0.32 (95% CI = 0.22 to 0.46).

**Table 3:** Associations of risk of malnutrition indicators with per 10 microgrammes per cubic metre (μg/m^3^) increase of ambient air pollution

	PM_2.5_	PM_c_	PM_10_
Main analysis*	**β (95% CI)**	**β (95% CI)**	**β (95% CI)**
Z-scores of HFA	-0.23 (-0.42,-0.05)†	-0.33(-0.58,-0.08)‡	-0.15(-0.26,-0.04)‡
Z-scores of WFA	0.18 (-0.02,0.38)	0.14 (-0.12,0.41)	0.09 (-0.03,0.21)
Z-scores of WFH	-0.09 (-0.33,0.15)	-0.05 (-0.37,0.27)	-0.04 (-0.19,0.10)
	**OR (95% CI)**	**OR (95% CI)**	**OR (95% CI)**
Stunting§	1.74 (1.11-2.72)†	2.96 (1.61-5.44)‖	1.51 (1.16-1.97)‡
Underweight¶	0.32 (0.22-0.46)‖	0.45 (0.28-0.71)‡	0.57 (0.47-0.71)‖
Wasting**	1.18 (0.65-2.16)	1.37 (0.59-3.16)	1.13(0.79-1.62)

PM_2.5_ – particulate matter with an aerodynamic diameter of 2.5 micron (μm); PM_c_ – particulate matter with an aerodynamic diameter of 2.5 to 10 μm, PM_10_ – particulate matter with an aerodynamic diameter of 10 μm, β – beta, CI – confidence interval, HFA – height for age, WFA – weight for age, WFH – weight for height, OR – odds ratio

*Main analysis: adjusted for age, sex, low birth weight, asthma history, anaemia history, history of dental caries, being ill for the last two weeks, optimal feeding scores, secondary smoke, residence, maternal education level, maternal height, maternal weight, mother suffering from anaemia during pregnancy, wealth category, drinking water source, relative humidity, mean temperature, altitude.

†*P*-value is between 0.01 and 0.05.

‡*P*-value is between 0.001 and 0.01.

§Stunting: Z-scores of HFA<-2.

‖*P* < 0.001.

¶Underweight: Z-scores of WFA<-2.

**Wasting: Z-scores of WFA<-2.

**Table 4** showed the effect estimates and 95% CI for the association between the malnutrition indicators and per IQR μg/m^3^ increase in average one-year PM_2.5_, PM_c_, and PM_10_ exposure according to the adjusted models. In general, PM_c_ and PM_2.5_ the maximum and minimum effect on Z-scores of HFA and stunting, respectively. For underweight, PM_2.5_ and PM_c_ showed the largest and smallest effect, respectively. For example, the results showed that the OR for stunting was 1.36 (95% CI = 1.06 to 1.75) per IQR μg/m^3^ increase in PM_2.5_, 1.80 (95% CI = 1.30 to 2.50) per IQR μg/m^3^ increase in PM_2.5_, and 1.55 (95% CI = 1.17 to 2.05) per IQR μg/m^3^ increase in PM_10_.

**Table 4:** Associations of risk of malnutrition indicators with per interquartile range (IQR) microgrammes per cubic metre (μg/m^3^) increase of ambient air pollution

	PM_2.5_	PM_c_	PM_10_
Main analysis*	**β (95% CI)**	**β (95% CI)**	**β (95% CI)**
Z-scores of HFA	-0.13 (-0.23,-0.03)†	-0.18 (-0.31,-0.04)‡	-0.16 (-0.27,-0.04)‡
Z-scores of WFA	0.10 (-0.01,0.21)	0.08 (-0.07,0.22)	0.10 (-0.03,0.22)
Z-scores of WFH	-0.05 (-0.19,0.08)	-0.03 (-0.20,0.14)	-0.05 (-0.20,0.10)
	**OR (95% CI)**	**OR (95% CI)**	**OR (95% CI)**
Stunting§	1.36 (1.06-1.75)†	1.80 (1.30-2.50)‖	1.55 (1.17-2.05)‡
Underweight¶	0.53 (0.43-0.64)‖	0.65 (0.50-0.83)‡	0.56 (0.45-0.69)‖
Wasting**	1.10 (0.79-1.54)	1.19 (0.75-1.87)	1.14 (0.77-1.67)

PM_2.5_ – particulate matter with an aerodynamic diameter of 2.5 μm, PM_c_ – particulate matter with an aerodynamic diameter of 2.5 to 10 μm, PM_10_ – particulate matter with an aerodynamic diameter of 10 μm, β – beta, CI – confidence interval, HFA – height for age, WFA – weight for age, WFH – weight for height, OR – odds ratio

*Main analysis: adjusted for age, sex, low birth weight, asthma history, anaemia history, history of dental caries, being ill for the last two weeks, optimal feeding scores, secondary smoke, residence, maternal education level, maternal height, maternal weight, mother suffering from anaemia during pregnancy, wealth category, drinking water source, relative humidity, mean temperature, altitude.

†*P-*value is between 0.01 and 0.05.

‡*P*-value is between 0.001 and 0.01.

§Stunting: Z-scores of HFA<-2.

‖*P* < 0.001.

¶Underweight: Z-scores of WFA<-2.

**Wasting: Z-scores of WFA<-2.

The relationships between long-term PM exposure and malnutrition indicators were nonlinear in the adjusted model (Figure S2-S7 in the **Online Supplementary Document**).

### Stratified analyses

**Figure 1:**
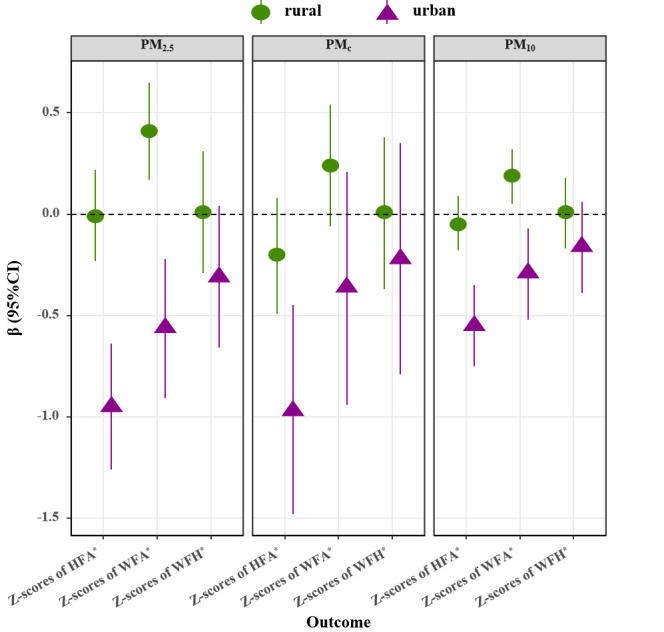
Associations of risk of continuous malnutrition indicators with per 10 microgrammes per cubic metre (μg/m^3^) increase of ambient air pollution stratified by residence. The adjusted models were adjusted for age, sex, low birth weight, asthma history, anaemia history, history of dental caries, being ill for the last two weeks, optimal feeding scores, secondary smoke, maternal education level, maternal height, maternal weight, mother suffering from anaemia during pregnancy, wealth category, drinking water source, relative humidity, mean temperature, altitude. *P value for difference <0.05. HFA – height for age, WFA – weight for age, WFH – weight for height

**Figure 2:**
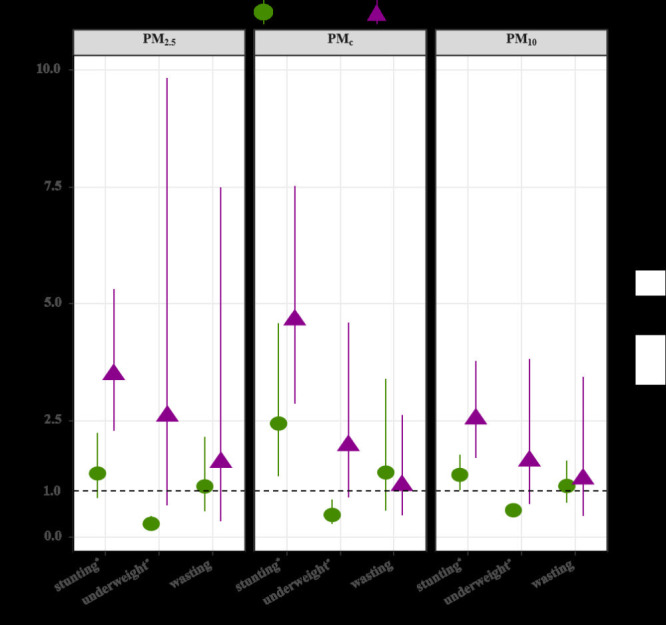
Associations of risk of categorical malnutrition indicators with per 10 microgrammes per cubic metre (μg/m^3^) increase of ambient air pollution stratified by residence. Stunting, Z-scores of HFA<-2; underweight: Z-scores of WFA<-2; wasting: Z-scores of WFA<-2; the adjusted models were adjusted for age, sex, low birth weight, asthma history, anaemia history, history of dental caries, being ill for the last two weeks, optimal feeding scores, secondary smoke, maternal education level, maternal height, maternal weight, mother suffering from anaemia during pregnancy, wealth category, drinking water source, relative humidity, mean temperature, altitude. *P value for difference <0.05.

**Figure 1** and **Figure 2** depicted the results of the stratified residence analyses for exposure to PM_2.5_, PM_c_, and PM_10_, respectively. In general, a greater effect of ambient PM was observed in urban areas. For Z-scores of HFA, Z-scores of WFA, Z-scores of WFH, stunting and underweight, the associations were stronger in urban areas than those in rural areas. For example, the association between stunting and per 10 μg/m^3^ increase in PM_2.5_ was significantly higher among urban children than rural children. As for wasting, the differences in effect estimations between the rural areas and urban areas were not significant.

Table S3-S8 in the **Online Supplementary Document** showed the results of the stratification analysis except for residence. In general, a greater effect of PM_2.5_ on Z-scores of HFA, Z-scores of WFA and Z-scores of WFH was observed in children who had low household income, unimproved drinking water sources, and low optimal feeding scores. For effect PM_2.5_ on stunting, there was no statistical difference between subgroups. The effect PM_2.5_ on underweight among children who were younger was greater. Similar effects also occurred in PM_c_ and PM_10_.

### Sensitivity analyses

Table S9-S12 in the **Online Supplementary Document** showed comparable effect estimates for malnutrition indicators when average ambient PM concentrations from different months before the survey were used as the exposure variable. For instance, increases of 10 μg/m^3^ in PM_2.5_ over nine months average concentration were associated with increases in the OR for stunting of 1.87 (95% CI = 1.11 to 3.16), the OR for underweight of 0.26 (95% CI = 0.17 to 0.39), the OR for wasting of 1.20 (95% CI = 0.59 to 2.42). The results from different exposure time indicated the robustness of the results.

## DISCUSSION

Long-term exposure to ambient PM_2.5_, PM_c_, and PM_10_ was associated with an increased risk of stunting, a decreased level of Z-scores of HFA, as well as a decreased risk of underweight in Tibet, with a greater effect observed in urban areas. To our knowledge, this is the first study to explore the urban-rural differences in the association between postnatal exposure to PM and six malnutrition indicators among children under five years old in China.

Our findings indicated long-term exposure to PM_2.5_, PM_c_, and PM_10_ were all positively associated with an increased risk of stunting. Few studies have addressed links between ambient air pollution and malnutrition indicators [14]. Only one study conducted in India found that exposure to 100 μg/m^3^ of PM_2.5_ in the month of birth was inversely associated with child HFA Z-score [20], which is in line with our results. Several biological mechanisms have been identified which could be responsible for the association between ambient air pollution and stunting. First, exposure to PM in early-life can adversely affect the development of immune function in children, contributing to recurrent illness [37]. For example, PM might impair linear growth through repeated episodes of febrile respiratory illness, which is associated with an increased risk of child stunting [38]. Besides, indirect route is possible, in which families divert income from food and nutrition to infection-related health costs, resulting in inadequate diets and impaired linear growth in children [39]. Second, children’s lungs are not fully formed until approximately six years of age. Repeated exposure to PM in young children might affect the structure and function of lung, triggering chronic immune activation, local and systemic inflammation, and growth hormone resistance [1]. A study has found chronic systemic inflammatory in children exposed to high concentrations of ambient PM [40]. Proinflammatory cytokines can directly affect growth through local regulation of chondrocytes [41]; in addition, proinflammatory cytokines can also combine with endocrine and nutritional factors to affect longitudinal bone growth by inhibiting insulin-like growth factor one [41]. Finally, air pollution might lead to vitamin D deficiency through multiple pathways, affecting immune function and bone metabolism [42]. Inadequate vitamin D concentrations are associated not only with an increased risk of respiratory infection in children, but also with bone metabolism and growth limitations [43,44].

The effects of ambient air pollution on stunting of urban children were found to be greater than those of rural children, which may be related to higher concentrations of ambient air pollution in urban areas than in rural areas. Our results indicated that the concentrations of ambient PM in urban areas of Tibet were higher than those in rural areas, which was consistent with some previous studies [45,46]. In addition, the PM concentrations and compositions between urban areas and rural areas were different [47]. This leads to different components of PM between urban and rural areas, as well as different toxicities of specific components in the missed composition, which in turn leads to different toxicities and health effects of PM [48].

The relationship between ambient PM and underweight has not yet been epidemiologically assessed among children under five years of age. A previous epidemiological study conducted in Nepal found that exposure to household air pollution was significantly associated with the risk of underweight among children aged 0-59 months [49]. We observed that long-term exposure to ambient PM_2.5_, PM_c_, and PM_10_ was negatively associated with an increased risk of underweight in Tibet. This inconsistency might be partly due to differences in PM concentration and composition across different study regions, and partly due to population variation [46]. Accumulating studies have shown that ambient PM is an important risk factor for obesity. Ambient PM may lead to insufficient physical activity and epigenetic modulation, promoting oxidative stress or inflammatory responses and subsequently increasing the risk of obesity [50,51]. Long-term exposure to ambient PM was associated with increased body weight, thereby protecting against underweight.

We found PM_2.5_ had the largest protective effect on underweight among the three PM fractions. Smaller particles could reach the depths of the respiratory tract, and had a higher surface volume ratio, and carried more toxins, thereby promoting more severe oxidative stress and inflammation [52]. Compared with PM_10_, PM_2.5_ contains a more complex mixture of fine particles and is more prone to obesity, which may explain the greater effect of PM_2.5_ on underweight. At the same time, we found that long-term exposure to PM_c_ had the greatest effect on the risk of stunting. The reason may be related to the different chemical composition, toxicity, and health effects of particulate pollutants with different particle sizes. In the future, research on the impact of different pollutants on children's growth and nutritional status needs to be further strengthened.

The stratification analysis showed that a greater effect of PM_2.5_ on Z-scores of HFA, Z-scores of WFA and Z-scores of WFH was observed in children who had low household income, unimproved drinking water sources, and low optimal feeding scores. The higher risk of low household income may be due to low household income inadequate diets for children and impaired linear growth [14]. As for unimproved drinking water source, it might be an important factor leading to diarrhoea and malnutrition [53]. Children with low optimal feeding scores may have difficulty in meeting their nutritional intake for growth and development.

There are several strengths in this study. First, to our knowledge, this is the first study to estimate the effect of postnatal exposure to air pollutants on six malnutrition indicators among children under five years old in mainland China. Second, we contrasted the effect of ambient PM with different particle sizes, which improved our understanding of the adverse effects of air pollutants on child malnutrition indicators. Third, we incorporated a rich set of covariates (feeding practice, secondary smoking exposure, water source, etc.) that have an important influence on the outcome to control for confounding issues in the analysis.

Some limitations of our study should be mentioned. First, our study only considered ambient air pollutants exposure, but not indoor air pollutants exposure, which is equivalent to treating all individuals as having the same indoor pollution level. Previous studies have found that indoor pollution has an impact on children's growth [18]. For those who were actually exposed to high indoor pollution (considered average indoor pollution) in this study, the results may overestimate the effect of outdoor air pollution on growth, i.e. the lower Z-scores of HFA in this sample may be attributable to indoor air pollution in addition to outdoor air pollution, or the combined effect of outdoor and indoor pollution. Second, recall bias may appear due to partial information (diet, disease history, etc.) were self-reported by respondents. Third, causal interpretations between air pollution exposures and malnutrition indicators should be made with caution considering the inherent limitation of cross-sectional design. Fourth, we averaged PM concentration across periods as individual exposure concentration, and did not fully account for seasonal differences in PM concentration and composition, which might have been the cause of risk of bias. Fifth, due to limited data availability, it was impossible to adjust all the potential confounding factors, such as occupation of guardian.

## CONCLUSIONS

Long-term exposure to PM_2.5_, PM_c_, and PM_10_ was associated with an increased risk of stunting and a decreased risk of underweight among children under five years old. And PM_c_ had the strongest association for stunting, while PM_2.5_ had the strongest association for underweight. Comparing rural areas, we observed the effects of ambient air PM exposure on the risk of malnutrition were pronounced in urban areas. Our findings supplement the limited evidence concerning the health hazard of ambient air pollution on child malnutrition indicators in low-pollution and high-altitude areas area of LMICs. More studies are needed to provide important scientific literature for understanding child malnutrition risk concerning postnatal exposure of air pollutants and formulating synthetically social and environmental policies for malnutrition prevention.

## Additional material


Online Supplementary Document
