# Supplementary material for: Urban-rural differences in the association between long-term exposure to ambient particulate matter (PM) and malnutrition status among children under five years old: A cross-sectional study in China
Source: J Glob Health. 2023 Sep 22;13:04112. doi: 10.7189/jogh.13.04112 (PMC10515095; doi:10.7189/jogh.13.04112)
Supplement: Online Supplementary Document [file jogh-13-04112-s001.pdf]

Supplementary material

Urban-rural differences in the association between long-term exposure to ambient particulate matter (PM) and malnutrition status among Tibetan children under five years old: a cross-sectional study in China

Contents

Figure S1 Flowchart of the study population.

Table S1 Association of risk of malnutrition indicators with per 10 μg/m^3 increase of ambient air pollution in crude models.

Table S2 Association of risk of malnutrition indicators with per IQR μg/m^3 increase of ambient air pollution in crude models.

Table S3 Association between $PM_{2.5}$, PM_c, and PM_{10} and Z-scores of HFA stratified by potential modifiers.

Table S4 Association between $PM_{2.5}$, PM_c, and PM_{10} and Z-scores of WFA stratified by potential modifiers.

Table S5 Association between $PM_{2.5}$, PM_c, and PM_{10} and Z-scores of WFH stratified by potential modifiers.

Table S6 Association between $PM_{2.5}$, PM_c, and PM_{10} and stunting stratified by potential modifiers.

Table S7 Association between $PM_{2.5}$, PM_c, and PM_{10} and underweight stratified by potential modifiers.

Table S8 Association between $PM_{2.5}$, PM_c, and PM_{10} and wasting stratified by potential modifiers.

Table S9 Association of risk of continuous malnutrition indicators with per 10 μg/m^3 increase of ambient air pollution in different exposure times.

Table S10 Association of risk of categorical malnutrition indicators with per 10 μg/m^3 increase of ambient air pollution in different exposure times.

Table S11 Association of risk of continuous malnutrition indicators with per IQR μg/m^3 increase of ambient air pollution in different exposure times.

Table S12 Association of risk of categorical malnutrition indicators with per IQR μg/m^3 increase of ambient air pollution in different exposure times.

Figure S2 β (solid red lines) and 95%CI (dashed lines) for Z-scores of HFA with ambient air pollutant exposure.

Figure S3 β (solid red lines) and 95%CI (dashed lines) for Z-scores of WFA with ambient air pollutant exposure.

Figure S4 β (solid red lines) and 95%CI (dashed lines) for Z-scores of WFH with ambient air pollutant exposure.

Figure S5 OR (solid red lines) and 95%CI (shaded part) for stunting with ambient air pollutant exposure.

Figure S6 OR (solid red lines) and 95%CI (shaded part) for underweight with ambient air pollutant exposure.

Figure S7 OR (solid red lines) and 95%CI (shaded part) for wasting with ambient air pollutant exposure.

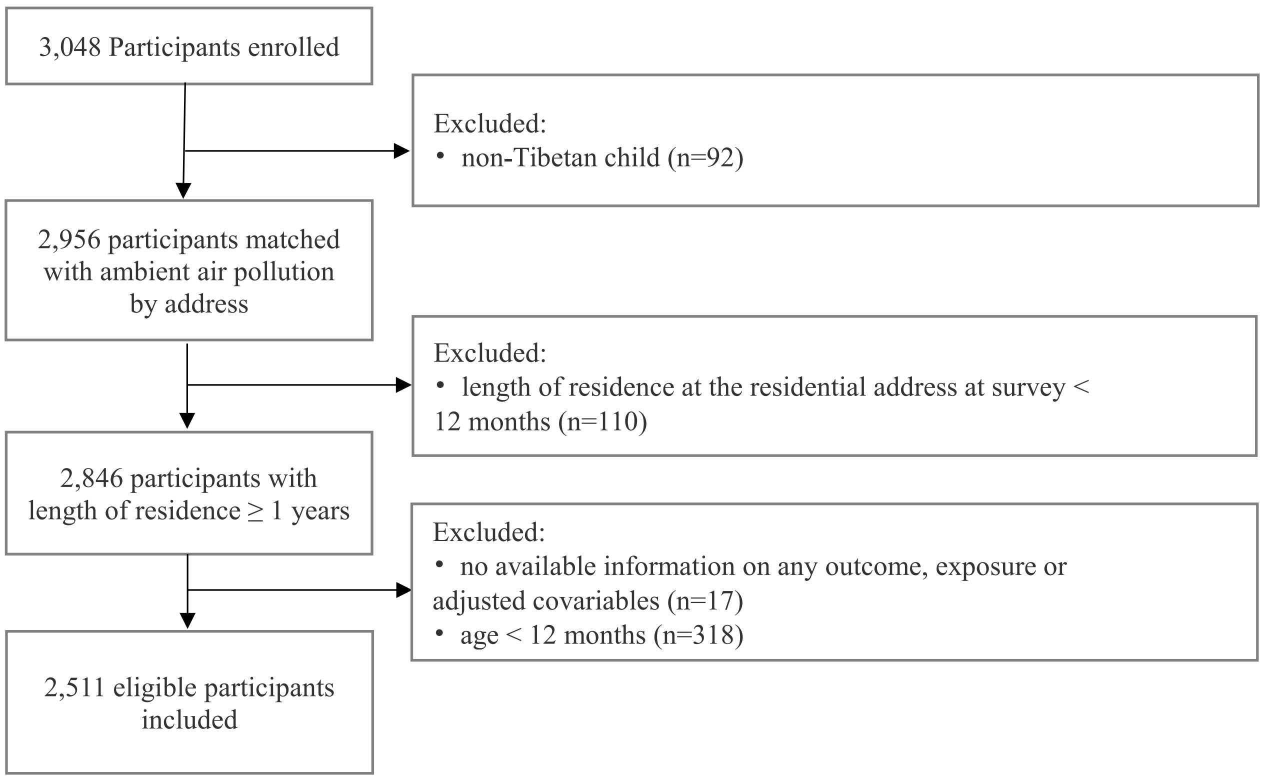

Figure S1 Flowchart of the study population.

Table S1 Associations of risk of malnutrition indicators with per 10 μg/m^3 increase of ambient air pollution in crude models.

	**$PM_{2.5}$**	**PM_c**	**PM_{10}**
Crude analysis[a]	**β(95%CI)**	**β(95%CI)**	**β(95%CI)**
**Z-scores of HFA**	**-0.45(-0.60, -0.30)*****	**-0.51(-0.67, -0.35)*****	**-0.26(-0.34, -0.18)*****
**Z-scores of WFA**	**-0.34(-0.50, -0.18)*****	**-0.18(-0.35, -0.01)***	**-0.14(-0.23, -0.06)****
**Z-scores of WFH**	**-0.26(-0.44, -0.08)****	**-0.20(-0.39, -0.01)***	**-0.12(-0.22, -0.03)***
	**OR(95%CI)**	**OR(95%CI)**	**OR(95%CI)**
**Stunting**[b]	**1.88(1.43, 2.48)*****	**3.01(2.17, 4.18)*****	**1.56(1.34, 1.82)*****
**Underweight**[c]	0.86(0.68, 1.08)	**0.75(0.59, 0.95)***	0.89(0.79, 1.00)
**Wasting**[d]	1.54(0.99, 2.39)	**1.80(1.09, 2.99)***	**1.31(1.03, 1.67)***

Notes: Bold fonts indicates the effect was statistically significant (P<0.05).

$PM_{2.5}$, particulate matter with an aerodynamic diameter of 2.5 μm; PM_c, particulate matter with an aerodynamic diameter of 2.5 to 10 μm, PM_{10} , particulate matter with an aerodynamic diameter of 10 μm.

HFA, height for age; WFA, weight for age; WFH, weight for height.

[a]Crude analysis: no adjustment.

[b]Stunting: Z-scores of HFA<-2.

[c]Underweight: Z-scores of WFA<-2.

[d]Underweight: Z-scores of WFA<-2.

***, P value <0.001; **, P value is between 0.001 and 0.01; *, P value is between 0.01 and 0.05.

Table S2 Associations of risk of malnutrition indicators with per IQR μg/m^3 increase of ambient air pollution in crude models.

	**$PM_{2.5}$**	**PM_c**	**PM_{10}**
Crude analysis[a]	**β(95%CI)**	**β(95%CI)**	**β(95%CI)**
**Z-scores of HFA**	**-0.25(-0.34, -0.17)*****	**-0.27(-0.36, -0.19)*****	**-0.27(-0.36, -0.19)*****
**Z-scores of WFA**	**-0.19(-0.28, -0.10)*****	**-0.10(-0.19, -0.01)***	**-0.15(-0.24, -0.06)****
**Z-scores of WFH**	**-0.15(-0.25, -0.04)****	**-0.11(-0.21, 0.00)***	**-0.13(-0.23, -0.03)***
	**OR(95%CI)**	**OR(95%CI)**	**OR(95%CI)**
**stunting**[b]	**1.42(1.22, 1.66)*****	**1.81(1.52, 2.17)*****	**1.60(1.37, 1.89)*****
**Underweight**[c]	0.92(0.81, 1.04)	**0.86(0.75, 0.97)***	0.88(0.77, 1.00)
**Wasting**[d]	1.27(0.99, 1.63)	**1.38(1.05, 1.81)***	**1.33(1.03, 1.72)***

Notes: Bold fonts indicates the effect was statistically significant (*P*<0.05).

$PM_{2.5}$, particulate matter with an aerodynamic diameter of 2.5 μm; PM_c, particulate matter with an aerodynamic diameter of 2.5 to 10 μm, PM_{10} , particulate matter with an aerodynamic diameter of 10 μm.

HFA, height for age; WFA, weight for age; WFH, weight for height.

[a]Crude analysis: no adjustment.

[b]stunting: Z-scores of HFA<-2.

[c]Underweight: Z-scores of WFA<-2.

[d]Underweight: Z-scores of WFA<-2.

***, P value <0.001; **, P value is between 0.001 and 0.01; *, P value is between 0.01 and 0.05.

Table S3 Association between $PM_{2.5}$, PM_c, and PM_{10} and Z-scores of HFA stratified by potential modifiers

Subgroup	$PM_{2.5}$		PM_c		PM_{10}	
	**β(95%CI)**	**P value for difference***	**β(95%CI)**	**P value for difference**	**β(95%CI)**	**P value for difference**
**Age, months**						
＜36	-0.35 (-0.85, 0.16)	Ref.	-0.81 (-1.39, -0.22)	Ref.	-0.31 (-0.59, -0.02)	Ref.
⩾36	-0.30 (-0.47, -0.12)	0.080	-0.36 (-0.60, -0.11)	**< 0.001**	-0.17 (-0.28, -0.07)	**< 0.001**
**Sex**						
Female	-0.44 (-0.70, -0.18)	Ref.	-0.49 (-0.84, -0.15)	Ref.	-0.25 (-0.41, -0.10)	Ref.
Male	-0.04 (-0.30, 0.22)	**< 0.001**	-0.18 (-0.54, 0.17)	**< 0.001**	-0.05 (-0.21, 0.11)	**< 0.001**
**Wealth status**						
High income	-0.26 (-0.52, 0.00)	Ref.	-0.23 (-0.60, 0.13)	Ref.	-0.14 (-0.30, 0.02)	Ref.
Low income	-0.27 (-0.54, -0.01)	0.462	-0.45 (-0.80, -0.11)	**< 0.001**	-0.19 (-0.34, -0.03)	**< 0.001**
**Optimal feeding scores**						
High scores	-0.18 (-0.53, 0.16)	Ref.	-0.39 (-0.81, 0.03)	Ref.	-0.15 (-0.35, 0.05)	Ref.
Low scores	-0.28 (-0.50, -0.05)	**< 0.001**	-0.38 (-0.70, -0.06)	0.823	-0.17 (-0.31, -0.04)	**0.042**
**Drinking water source**						
Improved	0.07 (-0.46, 0.59)	Ref.	-0.64 (-1.52, 0.23)	Ref.	-0.07 (-0.41, 0.27)	Ref.
Unimproved	-0.31 (-0.50, -0.11)	**< 0.001**	-0.31 (-0.56, -0.06)	**< 0.001**	-0.17 (-0.28, -0.06)	**< 0.001**

Notes: Bold fonts indicates the effect was statistically significant (*P*<0.05).

HFA, height for age.

The models were adjusted for age, sex, low birth weight, asthma history, anemia history, history of dental caries, being ill for the last two weeks, optimal feeding scores, secondary smoke, residence, maternal education level, Maternal height, maternal weight, mother suffering from anemia during pregnancy, wealth category, drinking water source, relative humidity, mean temperature, altitude.

*P value for difference: Z test was used to test for statistically significant difference in β estimates across categories within subgroups.

For example, in rural area vs urban area, we calculated: $Z=\frac{|\beta_{urban}-\beta_{rural}|}{\sqrt{se_{urban}^2+se_{rural}^2}}$.

Table S4 Association between $PM_{2.5}$, PM_c, and PM_{10} and Z-scores of WFA stratified by potential modifiers

Subgroup	$PM_{2.5}$		PM_c		PM_{10}	
	**β(95%CI)**	**P value for difference***	**β(95%CI)**	**P value for difference**	**β(95%CI)**	**P value for difference**
**Age, months**						
<36	0.00 (-0.51, 0.52)	Ref.	-0.51 (-1.10, 0.08)	Ref.	-0.12 (-0.41, 0.17)	Ref.
⩾36	0.12 (-0.10, 0.34)	**< 0.001**	0.54 (0.23, 0.85)	**< 0.001**	0.14 (0.01, 0.28)	**< 0.001**
**Sex**						
Female	0.00 (-0.29, 0.28)	Ref.	-0.06 (-0.43, 0.31)	Ref.	-0.01 (-0.18, 0.15)	Ref.
Male	0.36 (0.08, 0.64)	**< 0.001**	0.38 (0.00, 0.76)	**< 0.001**	0.20 (0.04, 0.37)	**< 0.001**
**Wealth status**						
High income	0.04 (-0.24, 0.32)	Ref.	0.11 (-0.28, 0.51)	Ref.	0.04 (-0.14, 0.21)	Ref.
Low income	0.23 (-0.05, 0.52)	0.462	0.12 (-0.25, 0.49)	0.827	0.10 (-0.06, 0.27)	**< 0.001**
**Optimal feeding scores**						
High scores	0.31 (-0.10, 0.72)	Ref.	0.34 (-0.15, 0.84)	Ref.	0.18 (-0.05, 0.41)	Ref.
Low scores	0.17 (-0.06, 0.40)	**< 0.001**	0.10 (-0.23, 0.42)	**< 0.001**	0.08 (-0.06, 0.22)	**< 0.001**
**Drinking water source**						
Improved	0.06 (-0.17, 0.30)	Ref.	0.14 (-0.16, 0.44)	Ref.	0.05 (-0.09, 0.19)	Ref.
Unimproved	0.34 (-0.09, 0.77)	**< 0.001**	-0.52 (-1.24, 0.20)	**< 0.001**	0.07 (-0.22, 0.35)	0.314

Notes: Bold fonts indicates the effect was statistically significant (*P*<0.05).
WFA, weight for age.
The models were adjusted for age, sex, low birth weight, asthma history, anemia history, history of dental caries, being ill for the last two weeks, optimal feeding scores, secondary smoke, residence, maternal education level, Maternal height, maternal weight, mother suffering from anemia during pregnancy, wealth category, drinking water source, relative humidity, mean temperature, altitude.
*P value for difference: Z test was used to test for statistically significant difference in β estimates across categories within subgroups.

For example, in rural area vs urban area, we calculated: $Z=\frac{|\beta_{urban}-\beta_{rural}|}{\sqrt{se_{urban}^2+se_{rural}^2}}$.

Table S5 Association between $PM_{2.5}$, PM_c, and PM_{10} and Z-scores of WFH stratified by potential modifiers

Subgroup	$PM_{2.5}$		PM_c		PM_{10}	
	β(95%CI)	P value for difference*	β(95%CI)	P value for difference	β(95%CI)	P value for difference
**Age, months**						
<36	0.31 (-0.34, 0.96)	Ref.	-0.11 (-0.85, 0.64)	Ref.	0.07 (-0.29, 0.44)	Ref.
⩾36	-0.21 (-0.44, 0.02)	**< 0.001**	-0.01 (-0.34, 0.31)	**0.021**	-0.08 (-0.22, 0.06)	**< 0.001**
**Sex**						
Female	-0.11 (-0.43, 0.21)	Ref.	-0.05 (-0.47, 0.37)	Ref.	-0.05 (-0.24, 0.14)	Ref.
Male	-0.08 (-0.44, 0.27)	0.250	-0.02 (-0.50, 0.46)	0.344	-0.03 (-0.25, 0.18)	0.270
**Wealth status**						
High income	-0.01 (-0.32, 0.29)	Ref.	-0.01 (-0.44, 0.42)	Ref.	-0.01 (-0.19, 0.18)	Ref.
Low income	-0.16 (-0.52, 0.20)	**< 0.001**	-0.11 (-0.57, 0.35)	**0.002**	-0.08 (-0.28, 0.13)	**< 0.001**
**Optimal feeding scores**						
High scores	-0.07 (-0.56, 0.43)	Ref.	0.11 (-0.49, 0.71)	Ref.	0.00 (-0.28, 0.29)	Ref.
Low scores	-0.05 (-0.32, 0.23)	0.468	-0.01 (-0.40, 0.38)	**0.002**	-0.02 (-0.18, 0.15)	0.234
**Drinking water source**						
Improved	-0.16 (-0.43, 0.12)	Ref.	-0.03 (-0.38, 0.32)	Ref.	-0.06 (-0.22, 0.10)	Ref.
Unimproved	-0.21 (-0.77, 0.36)	0.123	-0.96 (-1.89, -0.02)	**< 0.001**	-0.23 (-0.60, 0.13)	**< 0.001**

Notes: Bold fonts indicates the effect was statistically significant (P<0.05).

WFH, weight for height.

The models were adjusted for age, sex, low birth weight, asthma history, anemia history, history of dental caries, being ill for the last two weeks, optimal feeding scores, secondary smoke, residence, maternal education level, Maternal height, maternal weight, mother suffering from anemia during pregnancy, wealth category, drinking water source, relative humidity, mean temperature, altitude.

*P value for difference: Z test was used to test for statistically significant difference in β estimates across categories within subgroups.

For example, in rural area vs urban area, we calculated: $Z=\frac{|\beta_{urban}-\beta_{rural}|}{\sqrt{se_{urban}^2+se_{rural}^2}}$.

Table S6 Association between $PM_{2.5}$, PM_c, and PM_{10} and stunting stratified by potential modifiers

Subgroup	$PM_{2.5}$		PM_c		PM_{10}	
	**OR(95%CI)**	**P value for difference***	**OR(95%CI)**	**P value for difference**	**OR(95%CI)**	**P value for difference**
**Age, months**						
＜36	2.69 (1.31, 5.52)	Ref.	6.54 (2.64, 16.20)	Ref.	2.13 (1.40, 3.24)	Ref.
⩾36	1.34 (0.72, 2.51)	0.154	2.09 (0.87, 5.01)	0.076	1.27 (0.88, 1.85)	0.073
**Sex**						
Female	1.72 (0.86, 3.44)	Ref.	1.89 (0.74, 4.84)	Ref.	1.38 (0.91, 2.08)	Ref.
Male	2.05 (1.12, 3.76)	0.710	5.40 (2.35, 12.43)	0.102	1.80 (1.25, 2.59)	0.339
**Wealth status**						
High income	1.56 (0.76, 3.20)	Ref.	2.65 (0.97, 7.26)	Ref.	1.42 (0.92, 2.19)	Ref.
Low income	1.79 (0.99, 3.25)	0.776	2.82 (1.29, 6.15)	0.926	1.52 (1.07, 2.15)	0.813
**Optimal feeding scores**						
High scores	1.73 (0.75, 4.00)	Ref.	4.06 (1.34, 12.28)	Ref.	1.62 (0.99, 2.66)	Ref.
Low scores	1.80 (1.04, 3.10)	0.942	2.95 (1.39, 6.25)	0.641	1.52 (1.10, 2.11)	0.835
**Drinking water source**						
Improved	1.94 (1.14, 3.31)	Ref.	2.58 (1.30, 5.13)	Ref.	1.53 (1.12, 2.09)	Ref.
Unimproved	1.28 (0.50, 3.25)	0.447	6.37 (1.35, 30.14)	0.297	1.50 (0.82, 2.77)	0.964

Notes: Bold fonts indicates the effect was statistically significant (P<0.05).
Stunting, Z-scores of HFA ≤-2.
The models were adjusted for age, sex, low birth weight, asthma history, anemia history, history of dental caries, being ill for the last two weeks, optimal feeding scores, secondary smoke, residence, maternal education level, Maternal height, maternal weight, mother suffering from anemia during pregnancy, wealth category, drinking water source, relative humidity, mean temperature, altitude.
*P value for difference: Z test was used to test for statistically significant difference in β estimates across categories within subgroups.

For example, in rural area vs urban area, we calculated: $Z=\frac{|OR_{urban}-OR_{rural}|}{\sqrt{se_{urban}^2+se_{rural}^2}}$.

Table S7 Association between $PM_{2.5}$, PM_c, and PM_{10} and underweight stratified by potential modifiers

Subgroup	$PM_{2.5}$		PM_c		PM_{10}	
	OR(95%CI)	P value for difference*	OR(95%CI)	P value for difference	OR(95%CI)	P value for difference
**Age, months**						
＜36	1.20 (0.45, 3.16)	Ref.	4.78 (1.47, 15.54)	Ref.	1.51 (0.88, 2.62)	Ref.
⩾36	0.37 (0.26, 0.53)	**0.027**	0.20 (0.13, 0.33)	**< 0.001**	0.52 (0.42, 0.64)	**< 0.001**
**Sex**						
Female	0.29 (0.17, 0.52)	Ref.	0.41 (0.20, 0.85)	Ref.	0.55 (0.40, 0.76)	Ref.
Male	0.32 (0.20, 0.51)	0.838	0.45 (0.24, 0.83)	0.846	0.57 (0.43, 0.75)	0.853
**Wealth status**						
High income	0.39 (0.22, 0.69)	Ref.	0.64 (0.30, 1.38)	Ref.	0.65 (0.47, 0.92)	Ref.
Low income	0.29 (0.18, 0.47)	0.434	0.39 (0.21, 0.71)	0.317	0.55 (0.42, 0.72)	0.416
**Optimal feeding scores**						
High scores	0.18 (0.08, 0.38)	Ref.	0.32 (0.14, 0.74)	Ref.	0.45 (0.30, 0.68)	Ref.
Low scores	0.35 (0.23, 0.54)	0.124	0.50 (0.28, 0.90)	0.396	0.60 (0.47, 0.77)	0.239
**Drinking water source**						
Improved	0.32 (0.21, 0.49)	Ref.	0.40 (0.24, 0.68)	Ref.	0.56 (0.44, 0.72)	Ref.
Unimproved	0.32 (0.14, 0.71)	0.996	1.60 (0.39, 6.61)	0.074	0.66 (0.39, 1.10)	0.600

Notes: Bold fonts indicates the effect was statistically significant (P<0.05).
Underweight, Z-scores of WFA ≤ -2.
The models were adjusted for age, sex, low birth weight, asthma history, anemia history, history of dental caries, being ill for the last two weeks, optimal feeding scores, secondary smoke, residence, maternal education level, Maternal height, maternal weight, mother suffering from anemia during pregnancy, wealth category, drinking water source, relative humidity, mean temperature, altitude.
*P value for difference: Z test was used to test for statistically significant difference in β estimates across categories within subgroups.

For example, in rural area vs urban area, we calculated: $Z=\frac{|OR_{urban}-OR_{rural}|}{\sqrt{se^2_{urban}+se^2_{rural}}}$.

Table S8 Association between $PM_{2.5}$, PM_c, and PM_{10} and wasting stratified by potential modifiers

Subgroup	$PM_{2.5}$		PM_c		PM_{10}	
	**OR(95%CI)**	**P value for difference***	**OR(95%CI)**	**P value for difference**	**OR(95%CI)**	**P value for difference**
**Age, months**						
＜36	0.68 (0.19, 2.50)	Ref.	1.55 (0.37, 6.56)	Ref.	0.99 (0.49, 2.00)	Ref.
⩾36	1.54 (0.74, 3.20)	0.285	1.35 (0.44, 4.14)	0.882	1.24 (0.79, 1.96)	0.594
**Sex**						
Female	1.71 (0.63, 4.66)	Ref.	2.61 (0.63, 10.89)	Ref.	1.45 (0.79, 2.66)	Ref.
Male	1.05 (0.48, 2.30)	0.454	1.07 (0.37, 3.13)	0.327	1.03 (0.64, 1.65)	0.382
**Wealth status**						
High income	1.01 (0.39, 2.61)	Ref.	1.07 (0.29, 3.97)	Ref.	1.02 (0.57,1.80)	Ref.
Low income	1.62 (0.72, 3.64)	0.456	2.12 (0.68, 6.58)	0.440	1.37 (0.84, 2.22)	0.437
**Optimal feeding scores**						
High scores	0.64 (0.13, 3.21)	Ref.	2.41 (0.34, 17.17)	Ref.	1.04 (0.42, 2.56)	Ref.
Low scores	1.40 (0.71, 2.73)	0.378	1.28 (0.49, 3.35)	0.571	1.18 (0.79, 1.78)	0.798
**Drinking water source**						
Improved	0.97 (0.48, 1.99)	Ref.	0.89 (0.35, 2.28)	Ref.	0.97 (0.63, 1.48)	Ref.
Unimproved	3.84 (0.91, 16.25)	0.095	2.82 (1.56, 5.10)	**0.004**	3.50 (1.23, 9.98)	**0.026**

Notes: Bold fonts indicates the effect was statistically significant (*P*<0.05).
Wasting, Z-scores of WFH ≤ -2.
The models were adjusted for age, sex, low birth weight, asthma history, anemia history, history of dental caries, being ill for the last two weeks, optimal feeding scores, secondary smoke, residence, maternal education level, Maternal height, maternal weight, mother suffering from anemia during pregnancy, wealth category, drinking water source, relative humidity, mean temperature, altitude.
*P value for difference: Z test was used to test for statistically significant difference in β estimates across categories within subgroups.

For example, in rural area vs urban area, we calculated: $Z=\frac{|OR_{urban}-OR_{rural}|}{\sqrt{se_{urban}^2+se_{rural}^2}}$.

Table S9 Association of risk of continuous malnutrition indicators with per 10 μg/m^3 increase of ambient air pollution in different exposure times

Ambient Particulate	β (95%CI)		
Exposure times	Z-scores of HFA	Z-scores of WFA	Z-scores of WFH
**$PM_{2.5}$**			
Three months	**-0.58 (-0.88, -0.29)**	0.00 (-0.32, 0.32)	-0.28 (-0.66, 0.10)
Six months	**-0.46 (-0.72, -0.19)**	0.11 (-0.18, 0.39)	-0.20 (-0.54, 0.14)
Nine months	**-0.26 (-0.47, -0.04)**	0.23 (0.00, 0.46)	-0.09 (-0.37, 0.19)
One years	**-0.23 (-0.42, -0.05)**	0.18 (-0.02, 0.38)	-0.09 (-0.33, 0.15)
**PM_c**			
Three months	**-0.43 (-0.62, -0.23)**	-0.02 (-0.23, 0.19)	-0.06 (-0.32, 0.19)
Six months	**-0.60 (-0.89, -0.32)**	-0.04 (-0.34, 0.27)	-0.02 (-0.38, 0.35)
Nine months	**-0.52 (-0.85, -0.18)**	0.08 (-0.28, 0.44)	0.01 (-0.42, 0.44)
One years	**-0.33 (-0.58, -0.08)**	0.14 (-0.12, 0.41)	-0.05 (-0.37, 0.27)
**PM_{10}**			
Three months	**-0.26 (-0.38, -0.14)**	-0.01 (-0.14, 0.12)	-0.07 (-0.23, 0.09)
Six months	**-0.30 (-0.45, -0.16)**	0.02 (-0.14, 0.18)	-0.06 (-0.25, 0.12)
Nine months	**-0.20 (-0.34, -0.06)**	0.11 (-0.04, 0.26)	-0.04 (-0.22, 0.15)
One years	**-0.15 (-0.26, -0.04)**	0.09 (-0.03, 0.21)	-0.04 (-0.19, 0.10)

Notes: Bold fonts indicates the effect was statistically significant (P<0.05).
PM2.5, particulate matter with an aerodynamic diameter of 2.5 μm; PMc, particulate matter with an aerodynamic diameter of 2.5 to 10 μm, PM10 , particulate matter with an aerodynamic diameter of 10 μm.
HFA, height for age; WFA, weight for age; WFH, weight for height.
The models were adjusted for age, sex, low birth weight, asthma history, anemia history, history of dental caries, being ill for the last two weeks, optimal feeding scores, secondary smoke, residence, maternal education level, Maternal height, maternal weight, mother suffering from anemia during pregnancy, wealth category, drinking water source, relative humidity, mean temperature, altitude.

Table S10 Association of risk of categorical malnutrition indicators with per 10-μg/m[3] increase of ambient air pollution in different exposure times

**Ambient Particulate**	**OR (95%CI)**		
**Exposure times**	**Stunting**	**Underweight**	**Wasting**
**$PM_{2.5}$**			
Three months	**2.87 (1.36, 6.08)**	**0.19 (0.11, 0.34)**	1.19 (0.43, 3.28)
Six months	**2.51 (1.31, 4.83)**	**0.21 (0.13, 0.36)**	1.25 (0.52, 3.01)
Nine months	**1.87 (1.11, 3.16)**	**0.26 (0.17, 0.39)**	1.20 (0.59, 2.42)
One years	**1.36 (1.06, 1.75)**	**0.53 (0.43, 0.64)**	1.10 (0.79, 1.54)
**PM_c**			
Three months	**2.39 (1.41, 4.04)**	**0.52 (0.36, 0.74)**	1.24 (0.61, 2.50)
Six months	**4.78 (2.39, 9.55)**	0.60 (0.35, 1.04)	1.66 (0.64, 4.33)
Nine months	**5.01 (2.29, 10.96)**	0.61 (0.32, 1.18)	1.69 (0.55, 5.19)
One years	**1.80 (1.30, 2.50)**	**0.65 (0.50, 0.83)**	1.19 (0.75, 1.87)
**PM_{10}**			
Three months	**1.67 (1.22, 2.30)**	**0.59 (0.47, 0.74)**	1.11 (0.73, 1.71)
Six months	**2.11 (1.46, 3.04)**	**0.55 (0.42, 0.73)**	1.23 (0.75, 2.01)
Nine months	**1.78 (1.27, 2.49)**	**0.52 (0.40, 0.69)**	1.18 (0.74, 1.87)
One years	**1.55 (1.17, 2.05)**	**0.56 (0.45, 0.69)**	1.14 (0.77, 1.67)

Notes: Bold fonts indicates the effect was statistically significant (P<0.05).
PM2.5, particulate matter with an aerodynamic diameter of 2.5 μm; PMc, particulate matter with an aerodynamic diameter of 2.5 to 10 μm, PM10 , particulate matter with an aerodynamic diameter of 10 μm.
The models were adjusted for adjusted for age, sex, low birth weight, asthma history, anemia history, history of dental caries, being ill for the last two weeks, optimal feeding scores, secondary smoke, residence, maternal education level, Maternal height, maternal weight, mother suffering from anemia during pregnancy, wealth category, drinking water source, relative humidity, mean temperature, altitude.
Stunting: Z-scores of HFA<-2.
Underweight: Z-scores of WFA<-2.
Wasting: Z-scores of WFA<-2.

Table S11 Association of risk of continuous malnutrition indicators with per IQR μg/m^3 increase of ambient air pollution in different exposure times

Ambient Particulate	β (95%CI)		
**Exposure times**	**Z-scores of HFA**	**Z-scores of WFA**	**Z-scores of WFH**
**$PM_{2.5}$**			
Three months	**-0.33 (-0.49, -0.16)**	0.00 (-0.18, 0.18)	-0.16 (-0.37, 0.06)
Six months	**-0.26 (-0.40, -0.11)**	0.06 (-0.10, 0.22)	-0.11 (-0.30, 0.08)
Nine months	**-0.14 (-0.26, -0.02)**	0.13 (0.00, 0.26)	-0.05 (-0.20, 0.11)
One years	**-0.23 (-0.42, -0.05)**	0.18 (-0.02, 0.38)	-0.09 (-0.33, 0.15)
**PM_c**			
Three months	**-0.23 (-0.34, -0.12)**	-0.01 (-0.13, 0.10)	-0.03 (-0.17 ,0.10)
Six months	**-0.33 (-0.48, -0.17)**	-0.02 (-0.18, 0.14)	-0.01 (-0.20, 0.19)
Nine months	**-0.28 (-0.46, -0.10)**	0.04 (-0.15, 0.24)	0.00 (-0.23, 0.24)
One years	**-0.33 (-0.58, -0.08)**	0.14 (-0.12, 0.41)	-0.05 (-0.37, 0.27)
**PM_{10}**			
Three months	**-0.28 (-0.41, -0.15)**	-0.01 (-0.15, 0.13)	-0.07 (-0.24, 0.09)
Six months	**-0.32 (-0.48, -0.17)**	0.02 (-0.14, 0.19)	-0.07 (-0.27, 0.13)
Nine months	**-0.21 (-0.36, -0.06)**	0.12 (-0.04, 0.28)	-0.04 (-0.23, 0.16)
One years	**-0.15 (-0.26, -0.04)**	0.09 (-0.03, 0.21)	-0.04 (-0.19, 0.10)

Notes: Bold fonts indicates the effect was statistically significant (P<0.05).
PM2.5, particulate matter with an aerodynamic diameter of 2.5 μm; PMc, particulate matter with an aerodynamic diameter of 2.5 to 10 μm, PM10 , particulate matter with an aerodynamic diameter of 10 μm.
HFA, height for age; WFA, weight for age; WFH, weight for height.
The models were adjusted for age, sex, low birth weight, asthma history, anemia history, history of dental caries, being ill for the last two weeks, optimal feeding scores, secondary smoke, residence, maternal education level, Maternal height, maternal weight, mother suffering from anemia during pregnancy, wealth category, drinking water source, relative humidity, mean temperature, altitude.

Table S12 Association of risk of categorical malnutrition indicators with per IQR-μg/m^3 increase of ambient air pollution in different exposure times

Ambient Particulate	OR (95%CI)		
Exposure times	Stunting	Underweight	Wasting
**$PM_{2.5}$**			
Three months	**1.81 (1.19, 2.75)**	**0.40 (0.29, 0.55)**	1.10 (0.62, 1.95)
Six months	**1.67 (1.16, 2.42)**	**0.42 (0.32, 0.56)**	1.14 (0.70, 1.85)
Nine months	**1.42 (1.06, 1.91)**	**0.47 (0.37, 0.59)**	1.10 (0.74, 1.64)
One years	**1.74 (1.11, 2.72)**	**0.32 (0.22, 0.46)**	1.18 (0.65, 2.16)
**PM_c**			
Three months	**1.60 (1.21, 2.13)**	**0.70 (0.57, 0.85)**	1.12 (0.77, 1.64)
Six months	**2.33 (1.60, 3.39)**	0.76 (0.57, 1.02)	1.32 (0.78, 2.21)
Nine months	**2.39 (1.57, 3.65)**	0.77 (0.54, 1.09)	1.33 (0.72, 2.44)
One years	**2.96 (1.61, 5.44)**	**0.45 (0.28, 0.71)**	1.37 (0.59, 3.16)
**PM_{10}**			
Three months	**1.73 (1.23, 2.42)**	**0.58 (0.45, 0.73)**	1.12 (0.71, 1.77)
Six months	**2.20 (1.49, 3.25)**	**0.53 (0.40, 0.71)**	1.24 (0.74, 2.09)
Nine months	**1.84 (1.28, 2.63)**	**0.50 (0.38, 0.67)**	1.19 (0.73, 1.95)
One years	**1.51 (1.16, 1.97)**	**0.57 (0.47, 0.71)**	1.13 (0.79, 1.62)

Notes: Bold fonts indicates the effect was statistically significant (P<0.05).
$PM_{2.5}$, particulate matter with an aerodynamic diameter of 2.5 μm; PM_c, particulate matter with an aerodynamic diameter of 2.5 to 10 μm, PM_{10} , particulate matter with an aerodynamic diameter of 10 μm.
The models were adjusted for adjusted for age, sex, low birth weight, asthma history, anemia history, history of dental caries, being ill for the last two weeks, optimal feeding scores, secondary smoke, residence, maternal education level, Maternal height, maternal weight, mother suffering from anemia during pregnancy, wealth category, drinking water source, relative humidity, mean temperature, altitude.
Stunting: Z-scores of HFA<-2.
Underweight: Z-scores of WFA<-2.
Wasting: Z-scores of WFA<-2.

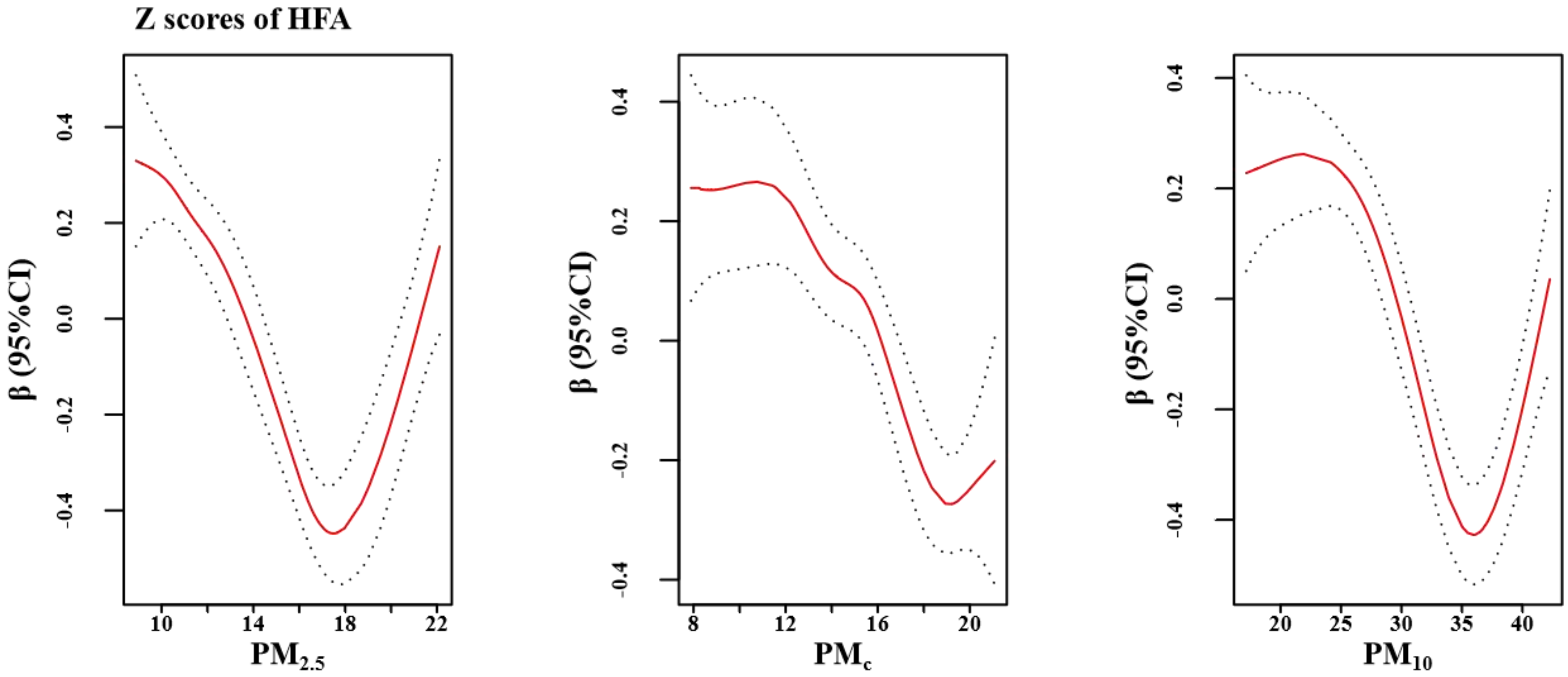

Figure S2 β (solid red lines) and 95%CI (dashed lines) for Z-scores of HFA with ambient air pollutant exposure.

Notes: HFA, height for age.

The adjusted models were adjusted for age, sex, low birth weight, asthma history, anemia history, history of dental caries, being ill for the last two weeks, optimal feeding scores, secondary smoke, residence, maternal education level, Maternal height, maternal weight, mother suffering from anemia during pregnancy, wealth category, drinking water source, relative humidity, mean temperature, altitude.

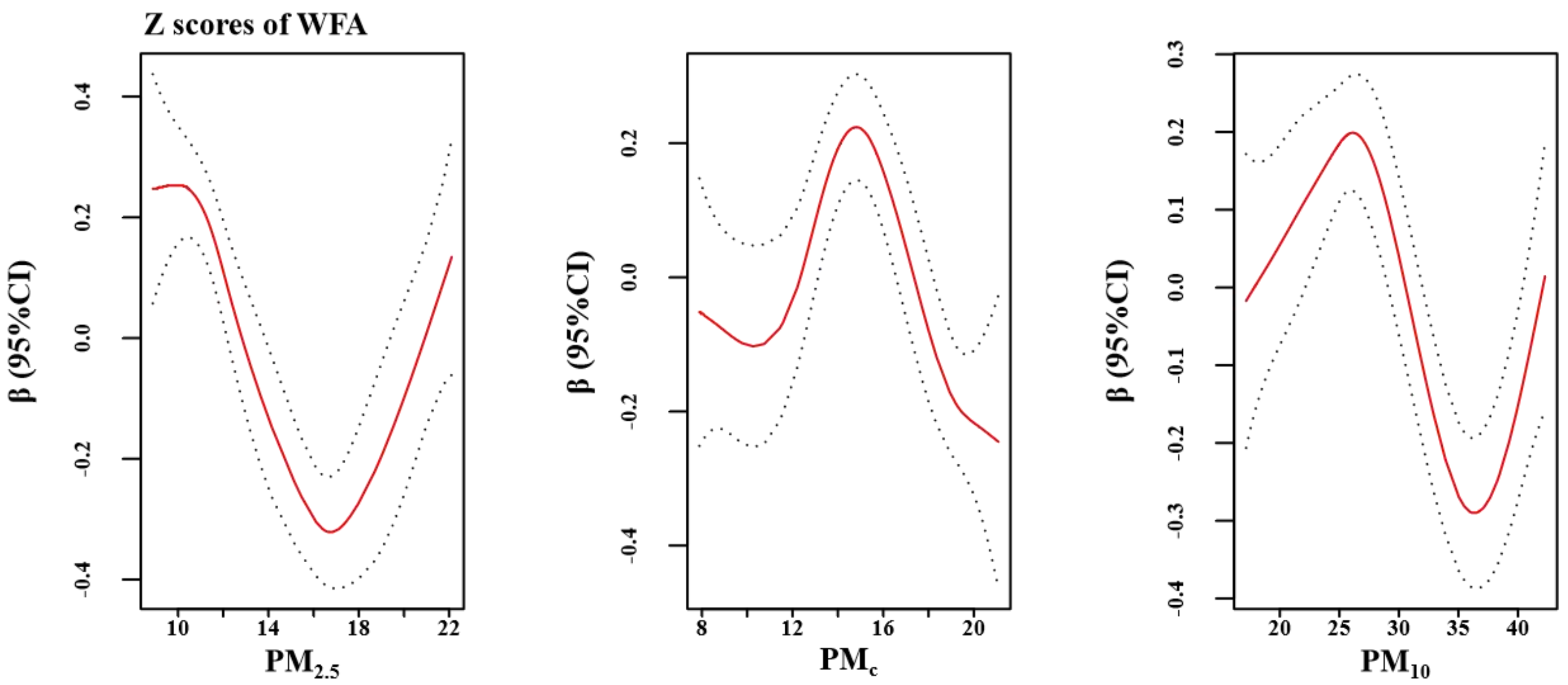

Figure S3 β (solid red lines) and 95%CI (dashed lines) for Z-scores of WFA with ambient air pollutant exposure.

Notes: WFA, weight for age.

The adjusted models were adjusted for age, sex, low birth weight, asthma history, anemia history, history of dental caries, being ill for the last two weeks, optimal feeding scores, secondary smoke, residence, maternal education level, Maternal height, maternal weight, mother suffering from anemia during pregnancy, wealth category, drinking water source, relative humidity, mean temperature, altitude.

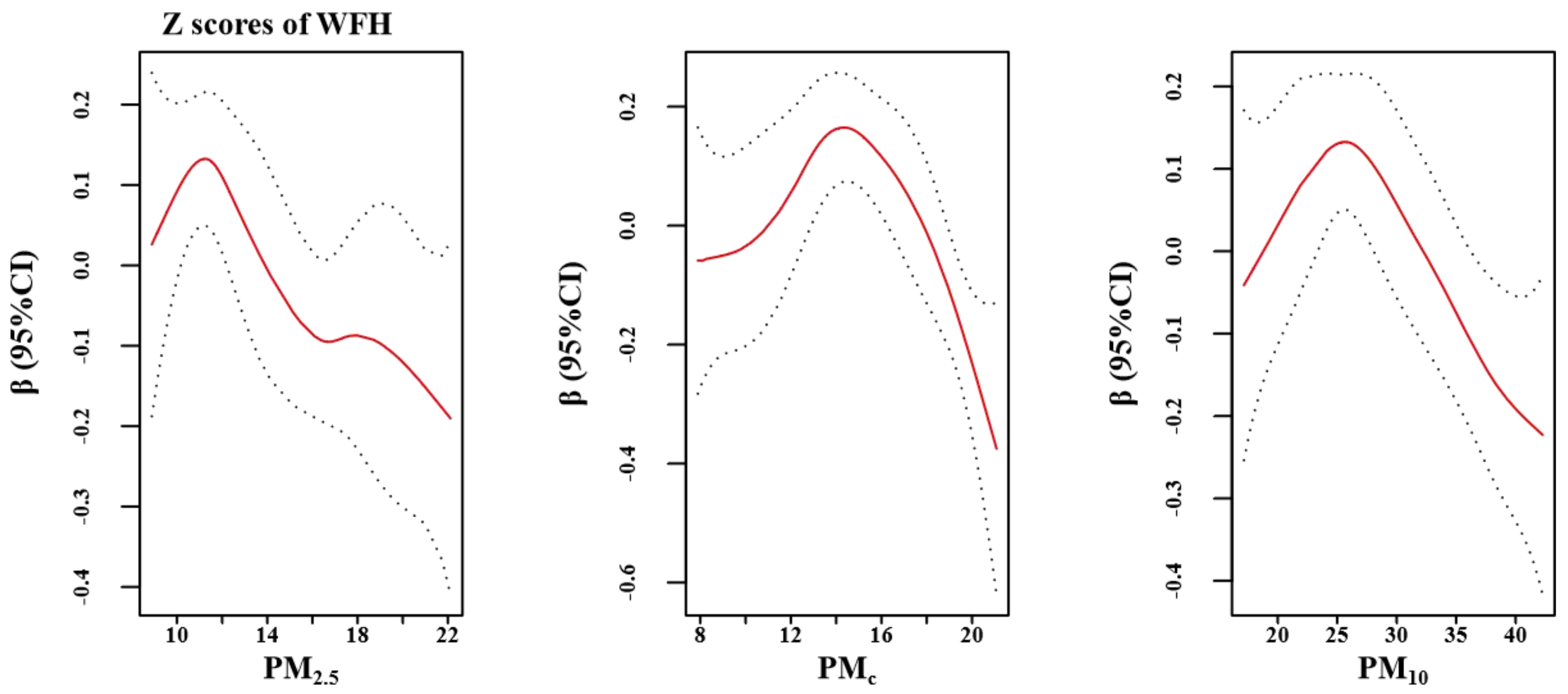

Figure S4 β (solid red lines) and 95%CI (dashed lines) for Z-scores of WFH with ambient air pollutant exposure.

Notes: WFA, weight for height.
The adjusted models were adjusted for age, sex, low birth weight, asthma history, anemia history, history of dental caries, being ill for the last two weeks, optimal feeding scores, secondary smoke, residence, maternal education level, Maternal height, maternal weight, mother suffering from anemia during pregnancy, wealth category, drinking water source, relative humidity, mean temperature, altitude.

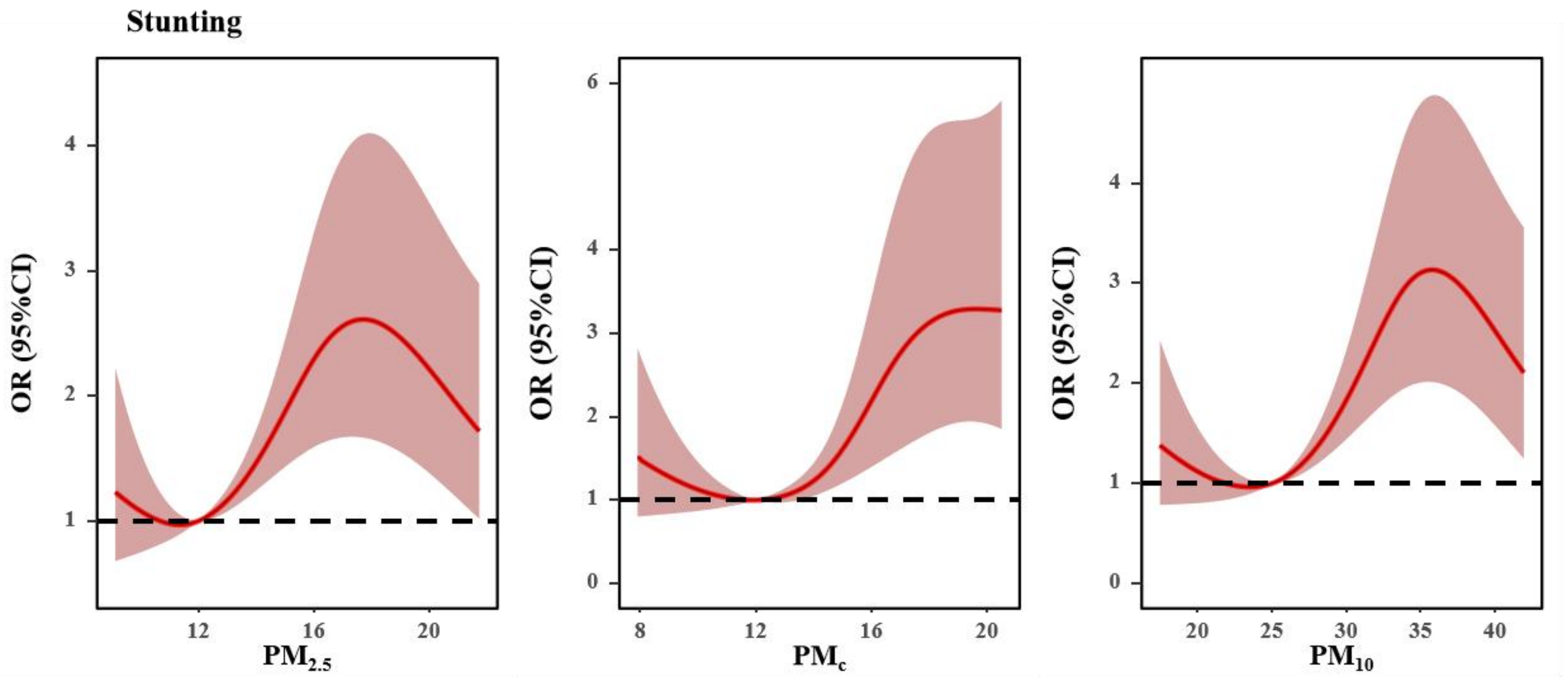

Figure S5 OR (solid red lines) and 95%CI (shaded part) for stunting with ambient air pollutant exposure.

Notes: Stunting: Z-scores of HFA<-2.

The adjusted models were adjusted for age, sex, low birth weight, asthma history, anemia history, history of dental caries, being ill for the last two weeks, optimal feeding scores, secondary smoke, residence, maternal education level, Maternal height, maternal weight, mother suffering from anemia during pregnancy, wealth category, drinking water source, relative humidity, mean temperature, altitude.

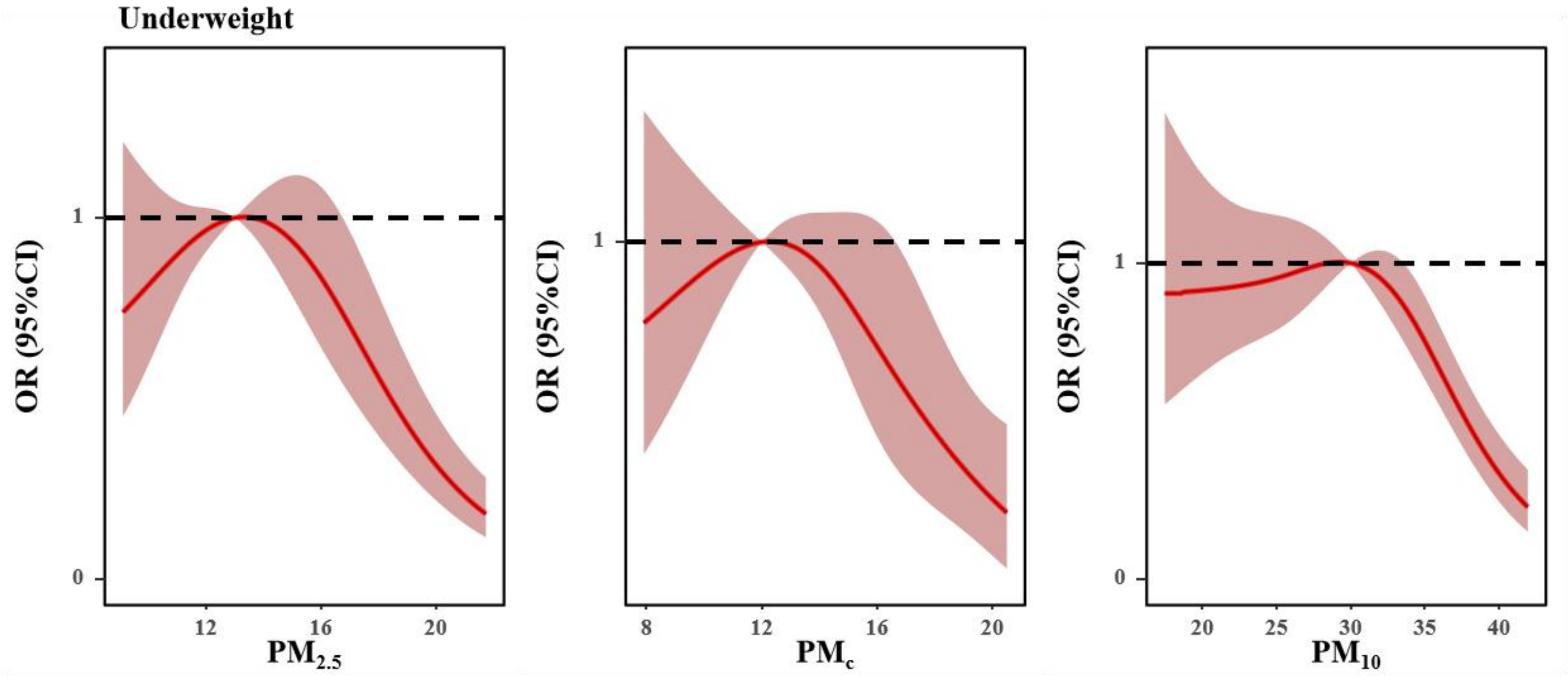

Figure S6 OR (solid red lines) and 95%CI (shaded part) for underweight with ambient air pollutant exposure.

Notes: Underweight: Z-scores of WFA<-2.

The adjusted models were adjusted for age, sex, low birth weight, asthma history, anemia history, history of dental caries, being ill for the last two weeks, optimal feeding scores, secondary smoke, residence, maternal education level, Maternal height, maternal weight, mother suffering from anemia during pregnancy, wealth category, drinking water source, relative humidity, mean temperature, altitude.

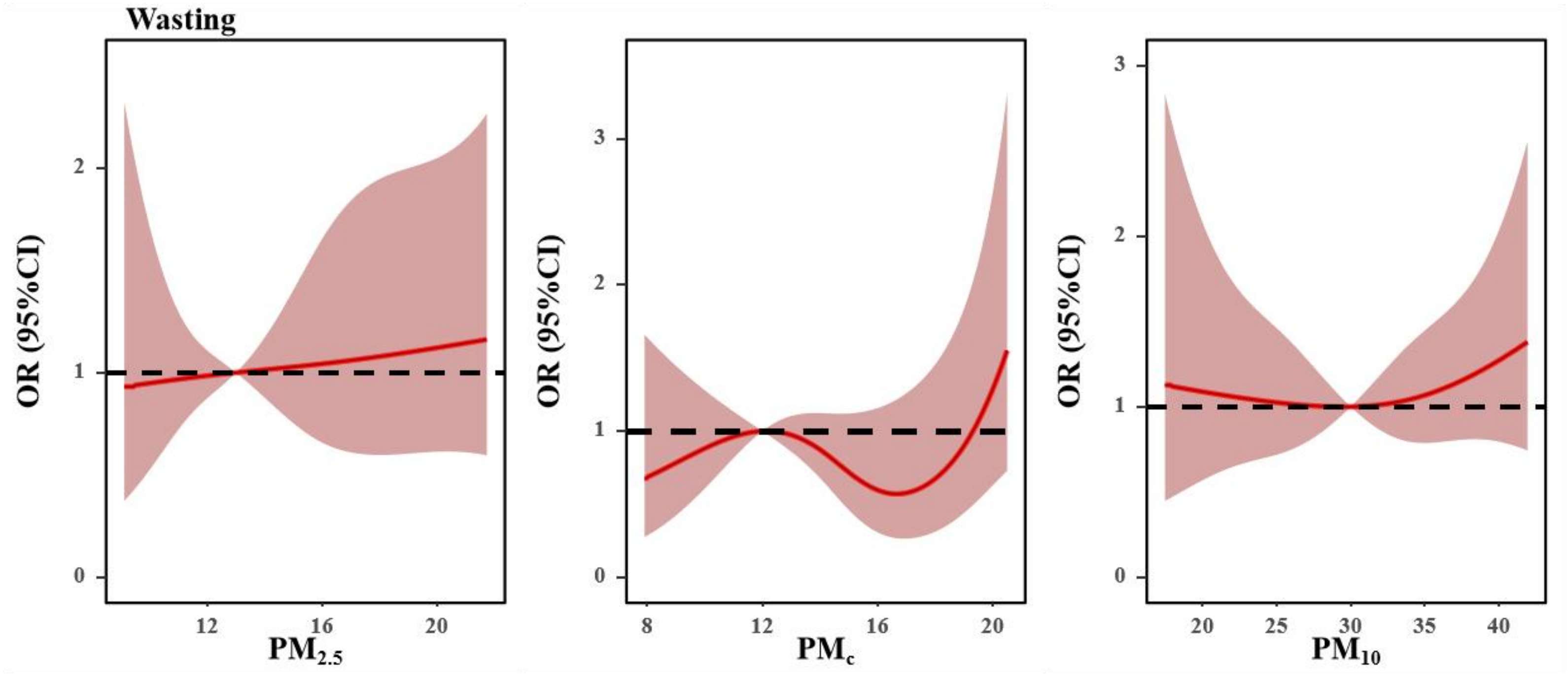

Figure S7 OR (solid red lines) and 95%CI (shaded part) for wasting with ambient air pollutant exposure.

Notes: Wasting: Z-scores of WFH<-2.

The adjusted models were adjusted for age, sex, low birth weight, asthma history, anemia history, history of dental caries, being ill for the last two weeks, optimal feeding scores, secondary smoke, residence, maternal education level, Maternal height, maternal weight, mother suffering from anemia during pregnancy, wealth category, drinking water source, relative humidity, mean temperature, altitude.
